# Chronic Stress Is Associated with Reduced Mindful Acceptance Skills but Not with Mindful Attention Monitoring: A Cross-Sectional Study

**DOI:** 10.3390/ijerph191811304

**Published:** 2022-09-08

**Authors:** Francesco Saldarini, Mark Cropley

**Affiliations:** School of Psychology, University of Surrey, Guildford GU2 7XH, Surrey, UK

**Keywords:** chronic stress, attention, acceptance, mindfulness, monitor and acceptance theory

## Abstract

Mindfulness-based interventions (MBIs) are effective in reducing chronic stress, but their therapeutic mechanisms are unclear. One possibility is that MBIs act by re-training attention monitoring and acceptance skills that have been impaired by chronic stress exposure. However, little research has investigated the association between chronic stress, monitoring, and acceptance. In this cross-sectional study we hypothesised observing correlations between stress, and (impaired) monitoring and acceptance. Moreover, we exploratively compared the magnitude of the correlations between chronic stress and four acceptance measures. Finally, we explored whether the association between stress and monitoring is moderated by acceptance. Eighty-five adults participated in the study and completed self-reported chronic stress and acceptance questionnaires and a mindful attention behavioural task. The results revealed that chronic stress was associated with reduced acceptance (all *p*s < 0.01) but not with monitoring. Exploratory analyses revealed no differences in the magnitude of the correlations between stress and each acceptance measure, except for the combined facets of mindfulness acceptance subscales and nonreactivity subscale (*p* = 0.023). Further analyses revealed a significant negative association between stress and the interaction between acceptance and the target detection component of monitoring (*p* = 0.044). Surprisingly, these results show that stress is associated with reduced monitoring at higher levels of acceptance. Theory-driven intervention studies are warranted to complement our results.

## 1. Introduction

Stress is a complex response to psychological (e.g., emotional abuse) or biological (e.g., threatening stimuli) challenges. Although reactions to stressors can be adaptive, chronic exposure to stress has been associated with maladaptive long-term changes in emotional, physiological, immune, and behavioural responses [1,2]. For example, stress has been proposed as a possible pathogenic mechanism for depression and anxiety [3,4,5]. There is a consistent body of research showing that mindfulness-based interventions (MBIs) are efficacious in reducing symptoms of stress [6], but little is known about their therapeutic mechanisms [7]. Monitor and acceptance theory (MAT) [8,9] is a recent psychobiological model that aims at explaining the therapeutic mechanisms of mindfulness-based interventions (MBIs) for stress. The theory proposes that MBIs reduce stress by training monitoring and acceptance skills.

Monitoring may be defined as the ability to maintain awareness of present-moment experience and may rely on the attentional neurocognitive subsystems of executive attention and orienting [9,10]. Existing research provides preliminary evidence supporting the role of mindfulness training in improving the performance and altering the neurophysiology of these two subsystems of attention [11,12,13,14]. Acceptance is an attitude of nonjudgement and equanimity toward any experience that may enter the field of awareness [9]. It is a form of emotion regulation strategy and is the opposite of avoiding or engaging in (stressful) thoughts or experiences. Instead, it consists in welcoming any experience into awareness until it eventually fades, as it is replaced by subsequent experiences. MBIs have consistently been shown to increase self-reported measures of acceptance [15]. Neuroimaging studies have also shown changes in brain activity in emotion-regulatory areas following mindfulness training (e.g., dorsolateral prefrontal cortex) [16].

MAT theorises that the practice of mindfulness trains the ability to monitor (i.e., notice, be aware of) present moment experiences and to regulate (distressing/emotional) experiences with acceptance. The interaction between monitoring and acceptance should drive stress reduction. This may imply that these functions are impaired in chronically stressed people and that “improvements” of these functions may underlie the positive effect of MBIs for stress reduction [8]. However, only preliminary evidence directly supports the idea that monitoring or acceptance are impaired in chronically stressed groups [17,18,19,20], or that the interaction between these two skills is associated with reduced chronic stress (or other health benefits) [21]. However, a vast literature shows that stress can generally impact cognition (e.g., working memory, interference inhibition) and emotion regulation, and vice-versa. Specifically, chronically released cortisol (i.e., a hormone secreted during the stress response) is known to bind several brain areas implicated in cognition and emotion and cause impairments in these functions [22,23]. Moreover, these brain areas are implicated in the regulation of the stress response and their impairment could contribute to maintaining a maladaptive chronic stress response [23,24,25]. Given this preliminary evidence, it is plausible to hypothesise that monitoring and acceptance are impaired in chronic stress groups compared to controls.

### 1.1. The Present Study

This theory-driven cross-sectional study aimed primarily at investigating the association of monitoring and acceptance measures with chronic stress. We hypothesised that self-reported measures of chronic stress are correlated with reduced attention monitoring efficiency (i.e., orienting and executive attention) and acceptance skills. We speculated that MBIs are effective in reducing stress in chronically stressed cohorts precisely because their monitoring and acceptance skills are impaired. Secondarily, this study aimed at exploring which specific acceptance variables show stronger correlations with chronic stress, as multiple self-reported measures of trait mindful acceptance exist. Although MAT broadly defines and operationalises acceptance [9], an acceptance measure showing a stronger correlation with chronic stress might be better suited to test MAT in the context of chronic stress studies compared to other scales. Moreover, we conducted these exploratory analyses to a priori identify a single measure of acceptance for our moderation analyses. Thirdly, this study aimed at exploring whether the interaction between monitoring and acceptance is associated with chronic stress. A significant moderation effect might provide preliminary support to MAT’s hypothesis that the interaction between monitoring and acceptance can drive stress reduction. We assessed self-reported levels of chronic stress using the perceived stress scale (PSS) [26] and assessed efficiency (i.e., reaction times) in orienting and executive control (i.e., monitoring) using the attentional network test (ANT) [27]. Finally, we measured acceptance skills with the five facets of mindfulness questionnaire (FFMQ—nonreactivity and nonjudgement subscales) [15] and Philadelphia mindfulness scale (PHLMS—acceptance subscale) [28]. To investigate the associations between variables (i.e., main hypothesis), we conducted correlational analyses between our chronic stress measure (i.e., PSS) and each monitoring and acceptance measure. To compare the magnitude of the correlations between our chronic stress measure and our acceptance measures (i.e., exploratory analysis 2), we statistically compared the correlation coefficients associating chronic stress with each of our acceptance measures. Measures showing stronger correlations with chronic stress might be employed in future studies testing MAT. To investigate the association between chronic stress and the interaction between monitoring and acceptance (i.e., exploratory analysis 2), we built two moderation models predicting chronic stress from acceptance, monitoring (i.e., orienting and target detection separately), and their interaction.

### 1.2. Novelty

This is a theory-driven study of specific hypotheses derived from MAT. Little theoretical research is generally conducted in psychology [29] and specifically on MAT [30], and, to our knowledge, only one other published study investigated theory-driven research questions similar to ours [21]. However, this research employed self-reported measures of monitoring, while we operationalised this variable with a behavioural task. The results of our study are complementary to existing MAT research and studies that serendipitously tested similar hypotheses with similar methods in other areas of psychology (e.g., [19]).

## 2. Materials and Methods

### 2.1. Design

This is a cross-sectional association study. The study was conceptualised, and this report written, according to the Strengthening the Reporting of Observational studies in Epidemiology guidelines for cross-sectional studies (STROBE) [31]. This study was pre-registered on the AsPredicted website (https://credlab.wharton.upenn.edu/ (accessed on 6 April 2021); see Supporting Information S1 for more details).

### 2.2. Participants

We invited 85 individuals from the general population to complete our questionnaires and behavioural measures. Advertisements and word of mouth were used to recruit participants from the University of Surrey and local area. Participants were included in the study if they were between 18 and 65 years old and had normal or corrected to normal vision. All participants were fluent in English. Participants provided written informed consent and received a GBP 7 shopping voucher or two university credits (if eligible) for their participation. The procedure was approved by the University of Surrey Ethics Committee and The Research Integrity and Governance Office (FHMS 20-21 159 EGA).

The sample size was determined a priori, with a power analysis conducted with the “pwr” package on R [32,33]. To compute sample size, we assumed a power of 0.8, and a significance level of *p* = 0.05 (two-tailed). Effect size was set at r = 0.3, as this should indicate a medium effect size. We defined r = 0.3 as the smallest effect size of interest, in the context of this study. We determined our effect size of interest based on Cohen’s heuristic, which defines a medium effect size as something visible to the naked eye of the careful observer [34,35] and the guidelines proposed by Lakens [36] who suggest calculating an experimental sample size based on the minimum effect size of interest. The results of our power analysis suggested a sample of 85 participants.

### 2.3. Procedure

Advertisements and word of mouth were used to recruit participants from the University of Surrey and local area. The advertisement campaign started in September 2021 and ended in March 2022. Potential volunteers contacted the experimenter via email, were screened according to our inclusion criteria, and then invited to participate in the study. On the day of the study, participants read an information sheet explaining the experimental procedure and signed a consent form. Volunteers who gave consent provided demographic data and completed three self-reported questionnaires and a behavioural task (see “Section 2.4” for more information). The order of the questionnaires (including demographics) and experimental task were randomised to prevent order-effect bias [37]. After completing the experiment, participants read a debriefing document containing additional information about the experiment, received compensation for their participation, and had the chance to ask for additional information. The entire procedure took approximately 50 min per participant to complete.

### 2.4. Measures and Variables

**Demographics:** Age (years), sex assigned at birth (male vs. female), education (years), and ethnicity (White, Black, Asian, Mixed, or Other (please specify)).

**Covariate**: Subjective level of current sleepiness was measured with the Karolinska Sleepiness Scale (KSS) [38]. This is a 9-point scale (1 = extremely alert, 2 = very alert, 3 = alert, 4 = rather alert, 5 = neither alert nor sleepy, 6 = some signs of sleepiness, 7 = sleepy—but no difficulty remaining awake, 8 = very sleepy—great effort to keep awake—fighting sleep, and 9 = extremely sleepy—fighting sleep). This sleepiness variable was used to control for sleepiness in our statistical analyses of the data obtained during the experimental tasks. We chose to control for sleepiness because performance in experimental tasks is known to deteriorate as a function of sleepiness [38]. The KSS is a valid measure of sleepiness [39].

**Chronic stress**: We obtained chronic stress measures using the perceived stress scale (PSS) [26], which was modified to assess perceived stress over the past 3 months [40]. Participants answered 14 questions on a Likert scale from 1 to 5, where 0 = never and 4 = very often. The PSS was designed to measure chronic stress in the general population along the dimensions of unpredictability, uncontrollability, and overload that relate to the risk for developing adverse health outcomes. It has strong construct validity and reliability across gender, socioeconomic status, age groups, ethnicity, and other demographic characteristics [26]. The PSS is widely used in studies investigating chronic stress [41] and was employed in one of the few existing studies of the association between monitoring and chronic stress [19].

**Acceptance**: We obtained scores in the acceptance subscales of the five facets of mindfulness questionnaire (FFMQ—nonreactivity and nonjudgement subscales) [15] and Philadelphia mindfulness scale (PHLMS—acceptance subscale) [28]. The FFMQ consists of 39 questions on a Likert scale from 1 to 5, where 1 = never or very rarely true and 5 = very often or always true. The two FFMQ subscales were considered as separate acceptance scales and the scores of each participant were summed to compute a third index of acceptance (i.e., FFMQ acceptance total). The PHLMS consist of 20 questions on a Likert scale from 1 to 5, where 1 = never and 5 = very often. The FFMQ and PHLMS were used in the research studies cited by Lindsay and Creswell [9], when defining acceptance in their mindfulness model. Therefore, these measures have a strong theoretical link to MAT. All questionnaires have good construct validity and reliability (FFMQ [15,42]; PHLMS [28]).

**Attention Monitoring**: We obtained performance scores (reaction times) in the orienting and target detection subscales of the attentional networks test (ANT) [27]. According to Lindsay and Creswell [9], monitoring relies on target detection and orienting, two key attentional subsystems [10,43]. The ANT was specifically designed to measure performance in these attentional subsystems. Moreover, the ANT was used in the few existing studies of the association between monitoring and chronic stress [19]. The duration of the ANT task is 25 min. It should be noted that the ANT can also measure performance in the third attentional subsystem of alerting. However, this subsystem is not theoretically linked to monitoring [9] and this data were not analysed in this study. The ANT task was programmed following the indications published by Fan et al. [27] and Westlye et al. [44]. The task was run on the Gorilla Experiment Builder platform (see the Section 3 for more information) and is an edited version of an open resource (https://app.gorilla.sc/openmaterials/50646 (accessed on 23 June 2022)).

### 2.5. Task Procedure

**Figure 1:**
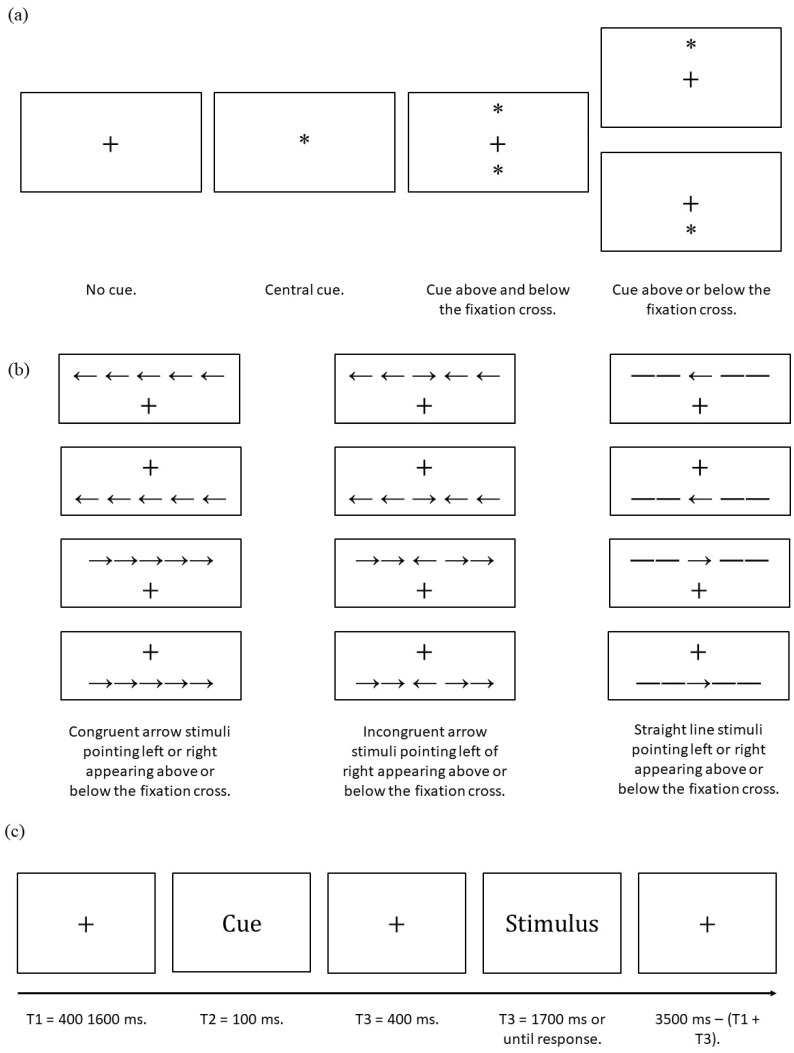
Schematic representation of the attentional networks test [27]; (**a**) all possible cues; (**b**) all possible stimuli; (**c**) timeline (ms = milliseconds; T = time).

The ANT requires participants to determine whether a central arrow presented in the stimuli phase points towards the left or right. The task consists of three experimental blocks each constituted of 96 trials. Each trial consists of five events. First a fixation cross is presented (random duration 400–1600 ms). Second, an asterisk cue appears (100 ms); this may be a central cue (i.e., appears in place of the fixation cross), no cue, double cue (i.e., two cues appear, one above and one below the fixation cross); or spatial cue (i.e., cue is above or below the fixation cross). The distance of the cues from the fixation cross is the same as the distance of the stimuli (see below) from the fixation cross; this does not include the fixation cue. The spatial cue condition provides spatial information about the upcoming stimuli location with 100% validity. Third, a second fixation cross is presented (400 ms). Then, stimuli are presented (1700 ms elapses or until the participants responds). Stimuli consists of a central arrow surrounded by four distractor flankers. Distractors may be straight lines (i.e., neutral condition) or arrows. Distractor arrows may point in the same or opposite direction of the central target arrow (i.e., congruent or incongruent trials respectively), and stimuli may appear above or below the fixation cross and the arrows may point either left or right. Finally, a third fixation cross (3500 ms minus duration of the first fixation cross minus reaction time in the stimulus phase). Therefore, the ANT has four cue conditions, two target locations, three flanker conditions, and two target directions (Figure 1). Each trial consists of a combination of these variables and is repeated twice per experimental block. Therefore, there are 96 trials per block.

Prior to the three experimental blocks, the task included two training blocks of 12 trials each. The first block presented a sample of 12 non-cued trials, while the second block presented a sample of 12 cued trials. Each block consisted of four congruent, four incongruent, and four neutral trials, balanced for arrow direction (i.e., left or right) and location (i.e., above or below the fixation cross). In the training blocks only, accuracy feedback was provided after response. Feedback consisted of a tick sign for correct responses (✓) and a cross mark for incorrect responses (×). The feedback signs appeared in the centre of the screen for 200 ms.

Arrow and line stimuli had an approximate size of 6.24 mm (i.e., 0.55° visual angle) and were separated by approximately 0.0681 mm (i.e., 0.06° visual angle). An array of five stimuli, including the separation between them, consisted of a total of approximately 3.950 cm (i.e., 3.08° visual angle). The distance between the fixation cross and stimuli array or asterisk was approximately 1.25 cm (i.e., 1.06° visual angle). All the above was designed in accordance with Fan et al. [27]. Details about the asterisk and fixation cross size were not specified in the original article by Fan et al.; therefore, we followed the guidelines offered by Westlye et al. [44]. Specifically, the fixation cross size was approximately 0.5 cm × 0.5 cm (i.e., 0.4407° × 0.4407° visual angle), while the asterisk diameter was approximately 0.3 cm (i.e., 0.2644° visual angle).

### 2.6. ANT Coefficients

To measure the efficiency in the orienting and target detection subsystems of attention, we recorded each participants’ reaction times (*RT*) in determining the direction of the target arrow during the stimulus phase. Then, we computed the mean *RT* for the central cue, spatial cue, incongruent trials, and congruent trials conditions. Finally, we computed our coefficients as follows:

*Orienting coefficient = Central cue RT − Spatial Cue RT*

*Target detection coefficient = Incongruent trials RT − Congruent trials RT*

Following the guidelines by Fan and Posner [45], Fan et al. [46], and Medina and Barraza [47], we interpreted higher orienting scores as more efficient compared to lower scores, and lower target detection scores as more efficient compared to higher scores. Only the reaction times associated with a correct response were considered in the analyses. Incorrect responses and omissions were excluded [27].

## 3. Apparatus

The experiment was presented on the Gorilla Experiment Builder platform [48] and was visualised on a DELL P1913 monitor with a 19 inches screen size, 1440 × 900 resolution, and 59 Hz refresh rate. Participants were positioned 65 cm away from the monitor.

## 4. Data Analysis

Data analyses were conducted with Python [49], the “cocor” R package [33,50], and Jamovi [51,52,53].

### 4.1. Preliminary Analyses

First, we explored demographic and self-reported measures using boxplots and tables, to identify and remove potential outliers. As for the behavioural measures, before computing the mean RT and ANT indices, the outliers outside the 1700 ms window (i.e., omissions or slow responses) were counted as incorrect responses and excluded from the analyses [54]. No further methods were used to exclude outliers. Second, we investigated whether our continuous data respected the assumptions of linearity required to conduct a correlation analysis. The assumption of normality was not investigated, as our sample was large enough to satisfy this assumption [55,56]. Third, we produced tables and boxplots to describe our data. Finally, as recommended by De Souza Almeida et al. [57], we reported reaction times and error rates for each ANT condition in table form (also see Fan et al. [27]; Kwak et al. [58]).

### 4.2. Main Analyses

We investigated the correlation of chronic stress (i.e., PSS) with monitoring (i.e., reaction times in each of the orienting and executive attention subsystems of attention) and acceptance (i.e., acceptance subscales of each mindfulness questionnaire and the sum of the two FFMQ subscales). All correlations that involved behavioural measures controlled for sleepiness, measured with the KSS. The significance of each r-value was tested with a two-tailed *t*-test. As we computed multiple correlation coeffects and their *p*-values, we increased the risk of type I error in our analyses. We used Bonferroni’s corrections to reduce this risk [59]. R^2^ values were computed, to investigate the amount of variance shared by each pair of correlated variables. Results were summarised in table and text form. All analyses dealt with potential missing data with a listwise deletion strategy.

### 4.3. Exploratory Analyses 1

The aim of these exploratory analyses was to compare the magnitude of the correlations between our chronic stress measure (i.e., PSS) and each acceptance measure we employed (i.e., FFMQ nonjudgement, FFMQ nonreactivity, FFMQ acceptance, and PHLMS acceptance). In doing so, we hoped to determine which of our acceptance measures varied the most as a function of chronic stress. The acceptance variable showing the strongest association with chronic stress might be employed in future studies investigating the effect of MBIs on chronic stress and acceptance. The statistical significance of the differences in magnitude between different overlapping and dependent correlation coefficients was tested using Dunn and Clark’s Z [60] and Zou’s confidence intervals [61].

### 4.4. Exploratory Analyses 2

The aim of these exploratory analyses was to investigate the interaction effect between monitoring and acceptance when predicting chronic stress (i.e., PSS scores). To do so, we built two moderation models (i.e., hierarchical multiple regression models with two step one predictors and the addition of an interaction effect between predictors at step two). The moderator predictor for both models was acceptance, operationalised as the combined scores of the two FFMQ acceptance subscales. We chose this as our measure of acceptance for these moderation analyses, because the scores in the combined FFMQ acceptance subscales had the strongest association with the PSS among the acceptance measures, in terms of absolute value (see the Section 5 for more information). Moreover, MAT does not differentiate between the two aspects of acceptance measured with the FFMQ subscales [9], which theoretically justifies the merging of the two measures. The second predictor differed in the two models. The first model included attention orienting (i.e., the first component of monitoring), operationalised as an orienting coefficient. The second model included target detection (i.e., the second component of monitoring), operationalised as a target detection coefficient. We followed up any significant interactions with a simple slope analysis. Even though these are exploratory analyses, we expected to observe significant moderation effects for our models. In addition, we expected the slopes of the simple slopes analyses to increase as a function of increases in the moderator. These predictions are in line with MAT.

## 5. Results

### 5.1. Preliminary Analyses

A total of 86 participants contacted the experimenter, were assessed for eligibility, and participated in the study. Due to a technical failure during data collection, data from one participant was lost, leaving us with a sample of 85 participants. Nine participants failed to report the number of years they spent in education, and four failed to report their age. The target detection coefficient could not be computed for three participants, as they failed to respond to all incongruent trials. These three participants’ data was removed in all analyses involving attention monitoring target detection. The exploration of our data with boxplots and summary tables did not reveal any outlying values in our acceptance or sleepiness measures. Our scatterplots of values of residuals against the value of outcomes predicted by our models did not reveal any patterns. Thus, our data satisfied the linearity assumption of correlational analysis (see the Supporting Information S2 for more details on preliminary analyses).

Descriptive statistics of our categorical variables show that our sample mostly consisted of female participants (69.4%). The sample consisted of participants who identified as White (64.7%), Asian (18.8%), Other (7.1%), Black (4.7%), or Mixed (4.7%). Descriptive statistics of our continuous variables are presented in Table 1. Reaction times and error rates for each ANT condition are reported in Table 2.

### 5.2. Main Analyses

Our results partially corroborated our hypotheses. As predicted, chronic stress was negatively associated with all mindful acceptance measures (i.e., all *p*-values < 0.001). These correlations remained significant after applying Bonferroni correction. The associations between chronic stress (i.e., PSS), nonjudgement and nonreactivity scores (i.e., FFMQ subscales) were accompanied by estimated medium effect sizes (i.e., all R^2^ > 0.13) [35]. The associations between chronic stress (i.e., PSS), acceptance total FFMQ, and PHLMS acceptance scores were accompanied by estimated large effect sizes (i.e., all R^2^ > 0.26). However, there was no significant correlation between chronic stress and our mindful attention measures. All associated effect sizes were trivial (i.e., all R^2^ < 0.001). The results from our analyses are reported in Table 3.

### 5.3. Exploratory Analyses 1

Our exploratory analyses revealed that our chronic stress measure (i.e., PSS) and the combined FFMQ subscales (i.e., FFMQ acceptance total) were significantly more negatively correlated than the PSS and the FFMQ nonreactivity subscale (i.e., *p* = 0.023; CI = [0.022, 0.307]). However, none of the other compared correlations statistically differed from one another. The results of our exploratory analyses are presented in Table 4.

### 5.4. Exploratory Analyses 2

We conducted preliminary analyses to ensure that our models respected the assumptions of multiple regression (see Supporting Information S3). Predictors were mean centred to reduce multicollinearity issues. We employed a combination of the FFMQ acceptance subscales as a measure of acceptance for these exploratory analyses. For completeness, and in the Supporting Information S4 we report the moderation analyses conducted, with all the alternative measures of acceptance we collected.

### 5.5. Model 1—Interaction between Orienting (i.e., Orienting Coefficient) and Acceptance (i.e., Combination of the FFMQ Acceptance Subscales)

At step one of our hierarchical multiple regression analysis, the two predictors (i.e., orienting and acceptance) explained 33% of the variance (R^2^ = 0.33 Adj. R^2^ = 0.31), which represented a statistically significant effect (*F* (2, 82) = 20.2; *p* < 0.001). However, the inclusion of the interaction term in step two did not contribute a statistically significant addition to the model (R^2^ *change* = 0.003; *F change* (1, 81) = 0.39; *p* = 0.53). This does not statistically support the presence of moderation. Table 5 below reports all the results for this model.

### 5.6. Model 2: Interaction between Target Detection (i.e., Target Detection Coefficient) and Acceptance (i.e., Combination of the FFMQ Acceptance Subscales)

At step one of our hierarchical multiple regression analysis, the two predictors (i.e., target detection and acceptance) explained 35% of the variance (R^2^ = 0.35 Adj. R^2^ = 0.33), which represented a statistically significant effect (*F* (2, 79) = 21.6; *p* < 0.001). The inclusion of the interaction term in step two contributed a statistically significant addition to the model (R^2^ *change* = 0.03; *F change* (1, 78) = 4.2; *p* = 0.044). This statistically supports the presence of moderation. The total variance explained by the model increased to 39% (R^2^ = 0.39 Adj. R^2^ = 0.36; *F* (3, 78) = 16.4; *p* < 0.001). Table 6 below reports all results for this model.

**Figure 2:**
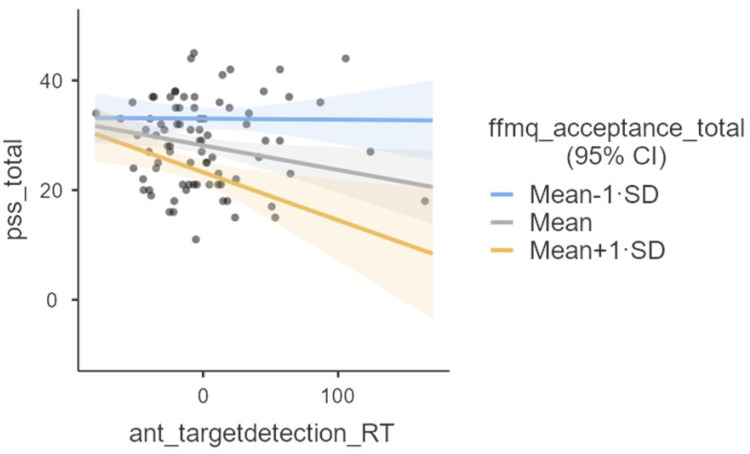
Visual representation of changes in the association between Chronic Stress (i.e., PSS total) and Target Detection (i.e., attention network test target detection reaction times [27]), as a function of Acceptance (i.e., five facets mindfulness questionnaire acceptance total [15]).

As the moderation effect was found to be significant, we followed up this result with simple slopes analyses. Table 7 reports the results for the simple slopes analysis, and Figure 2 provides a visual representation of these analyses.

## 6. Discussion

This study tested the hypothesis that chronic stress is associated with impaired mindful attention monitoring and acceptance skills, as defined in the MAT by Lindsay and Creswell [9]. Our results partially support this hypothesis. Specifically, self-reported chronic stress, measured with the PSS, was significantly negatively associated with all the self-reported acceptance measures. The effect sizes of these associations were medium or large, with the strongest association and largest effect size, in terms of absolute values, observed between PSS and the composite scale obtained by merging the two acceptance subscales of the FFMQ (i.e., FFMQ acceptance total). These results may be explained by hypothesising that people reporting higher levels of chronic stress may find MBIs helpful [6] precisely because these interventions train their ability to regulate emotions via acceptance. Improvement in acceptance, a possible emotion regulation mechanism [62], may then help regulate stress levels [7] and produce the observed health benefits. Future studies could empirically test this hypothesis by means of randomized controlled trials investigating whether specific changes in acceptance, as defined by Lindsay and Creswell [9], in people reporting higher levels of chronic stress are associated with or mediate reduced chronic stress, following an MBI.

Contrary to the predictions, our results show that self-reported chronic stress was not associated with either of the two components of mindful attention monitoring and the absolute effect sizes of these associations were trivial. Despite this, it should be noted that the confidence intervals extending from the *r* effect size indicator were rather large for both monitoring components, spanning from approximately −0.2 to +0.2. This might mean that some less trivial association might exist between these constructs. However, even considering the extremes of these confidence intervals, the effect sizes of the associations between chronic stress, orienting, and target detection, appear to be small. Thus, our results do not support our speculation that MBIs may act by training impaired monitoring skills in people reporting high levels of stress. However, it may still be hypothesised that MBIs promote stress reduction by improving monitoring skills above and beyond “normative/healthy” levels in people that do not show impaired monitoring skills, but report high chronic stress levels. Preliminary support for this alternative hypothesis is given by a vast literature documenting altered or improved cognition in meditators compared to healthy non-meditators (e.g., [63]). Alternatively, it could be hypothesised that chronic stress impairs cognition and emotion, but this effect cannot be observed behaviourally, only physiologically. Preliminary support for this alternative hypothesis is given by several publications reporting that people with stress-related illnesses (e.g., affective disorders) show differential brain activity, but not behavioural performance, during cognitive tasks, compared to controls (e.g., [64]). Finally, it may also be hypothesised that we did not observe the predicted results because of our theoretical or operational definition of monitoring. For example, monitoring may be a function of working memory, instead of attention, as defined by Petersen and Posner [10]. For a review on the relationship between working memory and mindfulness training see Jha et al. [65]. Future studies may further investigate the association between chronic stress and attention monitoring, adding to the limited existing literature.

We conducted exploratory analyses to follow up the observed negative correlations between PSS and the acceptance scales and tried to determine differences in magnitude between these associations. The results of these analyses showed that the correlation between chronic stress and the composite scale—obtained by merging the two acceptance subscales of the FFMQ (i.e., FFMQ acceptance total)—was larger than the correlation between chronic stress and FFMQ nonreactivity. This may imply that FFMQ acceptance total may be a more sensitive measure of change in acceptance compared to FFMQ nonreactivity, in the context of chronic stress. However, none of the other statistical comparisons showed differences in magnitude between correlations. This may imply that any of these scales may be equally employed to successfully detect changes in acceptance skills, in the context of chronic stress studies.

We conducted exploratory analyses to investigate whether the relationship between chronic stress (i.e., PSS scores) and monitoring (i.e., orienting and target detection separately) is moderated by acceptance (i.e., the summation of the scores of the FFMQ acceptance subscales). According to MAT, our results show that the relationship between target detection and chronic stress is moderated by acceptance. The effect size of the interaction appeared to be statistically small. However, contrary to MAT’s prediction, the association between the target detection coefficient (note that the larger this coefficient, the more inefficient the network [45]) and chronic stress became more negative as a function of acceptance. Thus, at higher levels of acceptance, inefficient target detection is associated with lower chronic stress. These unexpected results are difficult to explain and might be due to random variability. For example, one possibility is that target detection scores might not be reflective of differences in attention efficiency, but of strategies in approaching the task which might have randomly influenced the performance of our participants [45]. For example, people reporting lower stress levels might have preferred accuracy to speed, while the other participants might have preferred to give fast responses. Nevertheless, it is also possible that contrary to MAT’s prediction, at higher levels of acceptance, less efficient target detection is associated with lower chronic stress. Contrary to MAT predictions, we did not find a statistically significant moderation effect for our second moderation model employing orienting as a predictor, instead of target detection. The effect sizes of this non-significant interaction were trivial. Future intervention, controlled studies employing a MBI might further investigate the association of the interaction between monitoring and acceptance, and chronic stress.

### Limitations

A possible limitation of this study is that we operationalised each one of our phenomena of interest only on one level of description. That is, monitoring was operationalised as behavioural performance, while acceptance and chronic stress were operationalised as self-reported questionnaire scores. Future studies may measure these phenomena on multiple levels of description. For example, acceptance may be measured with questionnaires and task-based neuroimaging [66]. A possible second limitation of our study is the sample size, which was calculated assuming a medium effect size of the effects we investigated. As the monitoring and moderation effects sizes (but not those related to the acceptance measures) were small, a very large sample size might have allowed us to find significant results. However, it is unclear whether finding significant results for such small effect sizes would be of practical or theoretical interest [67].

## 7. Conclusions

This is one of the few theory-driven studies of specific hypotheses derived from MAT (e.g., [21,30]). MAT theorises that mindfulness training enhances the ability to monitor and accept present moment experiences and that the interaction between these abilities should be associated with reduced stress. Moreover, monitoring and acceptance should be impaired in chronically stressed people, and the effects of mindfulness training should be more evident in this population, compared to low-stress individuals. This study partially supports MAT’s theory, as it showed in our sample that chronic stress was associated with lower acceptance levels, but not with monitoring skills. Furthermore, after selecting the acceptance measure with the strongest correlation with stress, this study showed that only the association between stress and target detection (i.e., one monitoring component) was moderated by acceptance. However, the direction of this association was opposite to that hypothesized and reported by the existing literature [9]. We hope that future theory-driven studies will further test MAT and complement our research and the existing literature. Systematically testing and corroborating MAT might eventually lead to a better comprehension of the therapeutic mechanisms of mindfulness interventions and subsequently inform clinical practice and intervention development. 

## Figures and Tables

**Table 1:** Continuous variable descriptive statistics with means (M), standard deviations (SD), and range.

			Range	
	M	SD	Minimum	Maximum
***Demographics***				
Education (years)	16.6	2.33	12	23
Age (years)	22.99	5.46	18	45
***Covariate***				
Sleepiness	4.16	1.72	1	8
***Chronic stress***				
PSS	28.49	7.79	11	45
***Acceptance***				
FFMQ nonjudgement subscale	22.79	6.63	8	39
FFMQ nonreactivity subscale	20.78	4.59	10	30
FFMQ acceptance total	43.56	9.07	20	61
PHLMS acceptance subscale	26.86	7.76	12	44
***Monitoring***				
Orienting	42.93	27.95	−18.69	105.44
Target detection	102.08	41	22.77	266.39

Note. PSS = perceived stress scale; FFMQ = five facets mindfulness questionnaire; PHLMS = Philadelphia mindfulness scale. The FFMQ acceptance total scores were computed by summing each participant’s nonreactivity and nonjudgement scores.

**Table 2:** Reaction times (RT) and accuracy mean and standard deviation (SD) in milliseconds (ms) for each ANT condition.

	Cue Type			
Congruency	None	Centre	Double	Up/Down
(a) Mean RT (ms) and SD:				
Congruent	600.97 (42.41)	555.02 (47.5)	549.96 (35.52)	517.77 (52.01)
Incongruent	686.83 (46.61)	671.75 (51.64)	660.62 (52.88)	608.07 (45.03)
Neutral	590.6 (47.43)	547.18 (48.21)	540.81 (38.01)	508.47 (33.62)
(b) Mean accuracy and SD:				
Congruent	2.11 (5.67)	1.47 (4.32)	1.08 (3.95)	1.47 (4.22)
Incongruent	12.5 (22.26)	15.88 (22.19)	14.36 (23.25)	11.91 (22.73)
Neutral	2.55 (5.65)	2.06 (5.017)	2.4 (4.97)	2.01 (5.14)

**Table 3:** Correlations (r), 95% confidence intervals (CI95%), *p*-values, and R^2^ values between each experimental variable and chronic stress.

	M	SD	*n*	r	CI95%	*p*-Value	R^2^
***Covariate***							
Sleepiness	4.16	1.72	85	-	-	-	-
***Chronic stress***							
PSS	28.49	7.79	85	-	-	-	-
***Acceptance***							
FFMQ nonjudgement subscale	22.79	6.63	85	−0.499	[−0.64, −0.32]	<0.001	0.249
FFMQ nonreactivity subscale	20.78	4.59	85	−0.416	[−0.58, −0.22]	<0.001	0.173
FFMQ acceptance total	43.56	9.07	85	−0.575	[−0.7, −0.41]	<0.001	0.33
PHLMS acceptance subscale	26.86	7.76	85	−0.552	[−0.68, −0.38]	<0.001	0.304
***Monitoring*** ^a^							
Orienting	42.93	27.95	85	−0.029	[−0.24, 0.19]	0.79	0.001
Target detection	102.08	41	82	−0.017	[−0.23, 0.2]	0.88	0

Note. FFMQ = five facets mindfulness questionnaire; PHLMS = Philadelphia mindfulness scale. The FFMQ acceptance total scores were computed by summing each participant’s nonreactivity and nonjudgement scores. ^a^ Correlations corrected for the covariate sleepiness.

**Table 4:** Comparison of the correlation coefficients, including Dunn and Clark’s z and Zou’s confidence intervals (CI).

Compared r Coefficients ^a^		Inter Correlation ^b^	z-Value	*p*-Value	Zou’s CI
r (FFMQ nonjudgement)	r (FFMQ nonreactivity)	0.283	−0.743	0.457	[−0.303, 0.136]
r (FFMQ nonjudgement)	r (FFMQ acceptance total)	0.875	1.662	0.096	[−0.015, 0.178]
r (FFMQ nonjudgement)	r (PHLMS acceptance)	0.739	0.803	0.422	[−0.078, 0.189]
r (FFMQ nonreactivity)	r (FFMQ acceptance total)	0.713	2.27	0.023	[0.022, 0.307]
r (FFMQ nonreactivity)	r (PHLMS acceptance)	0.277	1.248	0.212	[−0.077, 0.352]
r (FFMQ acceptance total)	r (PHLMS acceptance)	0.681	−0.329	0.742	[−0.165, 0.117]

Note. FFMQ = five facets mindfulness questionnaire; PHLMS = Philadelphia mindfulness scale. The FFMQ acceptance total scores were computed by summing each participant’s nonreactivity and nonjudgement scores. ^a^ Each compared correlation coefficient (i.e., r) is the association between the PSS (perceived stress scale) and the acceptance measure specified in parentheses. ^b^ Association between the non-overlapping measures (i.e., the acceptance measures) used to compute the r-values compared in this table.

**Table 5:** Moderation model results for the prediction of Chronic Stress (i.e., PSS score) from Orienting (i.e., orienting coefficient) moderated by Acceptance (i.e., combination of the FFMQ acceptance subscales score).

			95% Confidence Intervals						
Predictor	Estimate (B)	SE	Lower	Upper	t	*p*	Standard Estimate (β)	r^2^ _a(b,c)_	R^2^ Change
Step 1 ^a^									0.33
Orienting	−0.0044	0.02528	−0.05469	0.0459	−0.174	0.862	−0.0158	0.000256	
FFMQ acceptance total	−0.48585	0.07867	−0.64238	−0.32932	−6.176	<0.001	−0.5661	0.3136	
Step 2 ^a^									0.00322
Orienting X FFMQ acceptance total	−0.00196	0.00312	−0.00817	0.00426	−0.626	0.533	−0.0573	0.003249	

Note. r^2^ _a(b,c)_ = semi-partial correlation of the dependent (i.e., PSS) with the predictor variables, controlled for the remaining two predictors. PSS = perceived stress scale. FFMQ = five facets mindfulness questionnaire. ^a^ Steps of the hierarchical multiple regression.

**Table 6:** Moderation model results for the prediction of Chronic Stress (i.e., PSS score) from Target detection (i.e., orienting coefficient) moderated by Acceptance (i.e., combination of the FFMQ acceptance subscales score).

			95% Confidence Intervals						
Predictor	Estimate (B)	SE	Lower	Upper	t	*p*	Standard Estimate (β)	r^2^ _a(b,c)_	R^2^ Change
Step 1 ^a^									0.354
Target detection	−0.04535	0.01898	−0.08314	−0.00756	−2.39	0.019	−0.237	0.044944	
FFMQ acceptance total	−0.52917	0.07811	−0.68468	−0.37366	−6.77	<0.001	−0.619	0.361201	
Step 2 ^a^									0.033
Target detection X FFMQ acceptance total	−0.00463	0.00226	−0.00913	−1.31 × 10^−4^	−2.05	0.044	−0.198	0.033124	

Note. r^2^ _a(b,c)_ = semi-partial correlation of the dependent (i.e., PSS) with the predictor variables, controlled for the remaining two predictors. PSS = perceived stress scale. FFMQ = five facets mindfulness questionnaire. ^a^ Steps of the hierarchical multiple regression.

**Table 7:** Simple slopes analyses showing changes in the association between Chronic Stress (i.e., PSS scores) and Target detection (i.e., target detection coefficient) as a function of Acceptance (i.e., combination of the FFMQ acceptance subscales scores).

Moderator Levels			95% Confidence Interval				
FFMQ Acceptance Total	Estimate	SE	Lower	Upper	df	t	*p*
Mean − 1·SD	−0.00179	0.0223	−0.0463	0.04268	78	−0.0802	0.936
Mean	−0.0446	0.0188	−0.0821	−0.00708	78	−2.3667	0.02
Mean + 1·SD	−0.0874	0.0329	−0.1529	−0.02186	78	−2.6547	0.01

## Data Availability

As the study participants did not consent to sharing their data, raw data are not publicly available. The experimental task and questionnaires, and data-cleaning and analysis procedures, can be found at: https://app.gorilla.sc/openmaterials/421858 or at https://osf.io/9zahq/ (accessed on 23 June 2022).

## Supplementary Materials

The following supporting information can be downloaded at: https://www.mdpi.com/article/10.3390/ijerph191811304/s1, Supporting Information S1: Study1_supporting information_v4; Supporting Information S2: Study1_supporting information_v4; Supporting Information S3: Study1_supporting information_v4; Supporting Information S4: Study1_supporting information_v4.
