# Supplementary material for: Chronic Stress Is Associated with Reduced Mindful Acceptance Skills but Not with Mindful Attention Monitoring: A Cross-Sectional Study
Source: Int J Environ Res Public Health. 2022 Sep 8;19(18):11304. doi: 10.3390/ijerph191811304 (PMC9517081; doi:10.3390/ijerph191811304)
Supplement: Supplementary file 1 [file ijerph-19-11304-s001.zip › ijerph-1839605-supplementary.pdf]

Chronic stress is associated with reduced mindful acceptance skills but not with mindful attention monitoring: a cross-sectional study.

Supporting information.

Pre-registration on AsPredicted. S1.

AsPredicted is an online research depository provided by the University of Pennsylvania, Wharton Credibility Lab (https://credlab.wharton.upenn.edu/). Our pre-registration will be made publicly available on this platform after the potential publication (or "pre-print") of our manuscript. It should be noted that our final manuscript presents a minor difference with the preregistration. That is, in the preregistration, we say that "All correlations will be controlled for sleepiness, measured with the Karolinska Sleepiness Scale (Gillberget al., 1994)". This was a mistake on our part. In fact, we wished to control only those correlations that involved a behavioural component. This is because the reason of the correction is that sleepiness in known to impact the performance in behavioural tasks (see the "*Measures and variables*" section in the main manuscript). Thus, it would make no sense to correct those correlations that only involved questionnaires.

Summary tables and boxplots produced to detect outliers. S2.

The exploration of our data with boxplots and summary tables did not reveal any outlying values (see the Supporting Information for more details) in our acceptance and sleepiness variables. A few potential outliers were detected in the years of education and age variables. As these variables were used only to describe our sample and the outlying values could not be attributed to incorrectly entered data, we did not exclude these outliers from our data set. Our

scatterplots of values of residuals against the value of outcomes predicted by our models did not reveal any pattern. Thus, our data satisfied the linearity assumption of correlational analysis. The tables and boxplots used to summarise our data, as well as the scatterplots used to explore the assumption of linearity, are reported in the Supporting Information.

DECRIPTIVE STATISTICS

Sex (male %) = 30.588
Education in years [mean (sd)] = 16.598684210526315(2.331) Range min: 12.0Range max: 23.0
Age in years [mean (sd)] = 22.987654320987655(5.456) Range min: 18.0Range max: 45.0
Sleepiness [mean (sd)] = 4.1647058823529415(1.721) Range min: 1.0Range max: 8.0
Ethnicity (white %) = 64.706
Ethnicity (black %) = 4.706
Ethnicity (asian %) = 18.824
Ethnicity (mixed %) = 4.706
Ethnicity (other %) = 7.059
PSS [mean (sd)] = 28.49411764705882(7.785) Range min: 11.0Range max: 45.0
FFMQ nonjudge [mean (sd)] = 22.788235294117648(6.633) Range min: 8.0Range max: 39.0
FFMQ nonreact [mean (sd)] = 20.776470588235295(4.585) Range min: 10.0Range max: 30.0
FFMQ acceptance total [mean (sd)] = 43.56470588235294(9.07) Range min: 20.0Range max: 61.0
PHLMS acceptance [mean (sd)] = 26.858823529411765(7.763) Range min: 12.0Range max: 44.0
ANT orienting RT [mean (sd)] = 42.930844984094115(27.949) Range min: -18.687107938532563Range max: 105.4437499999865
ANT target detection RT [mean (sd)] = 102.08418855568142(41.002) Range min: 22.773913043525795Range max: 266.3912364103548

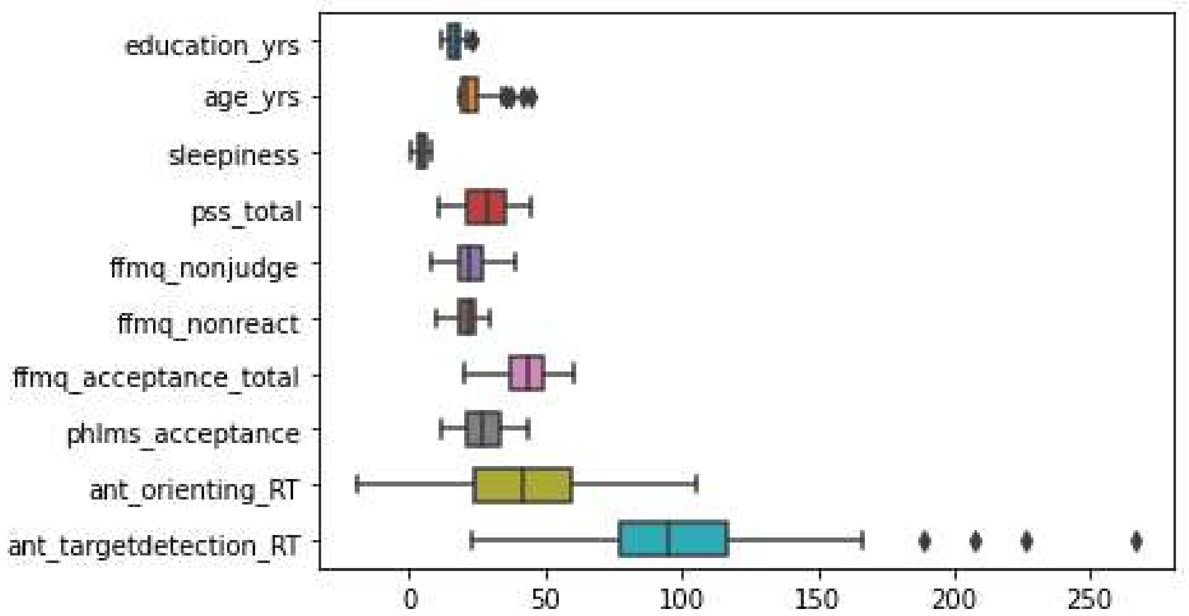

In the boxplots, outliers are defined as Q1 - 1.5 * IQR and Q1 + 1.5 * IQR.

Scatterplots used to explore the assumption of linearity.

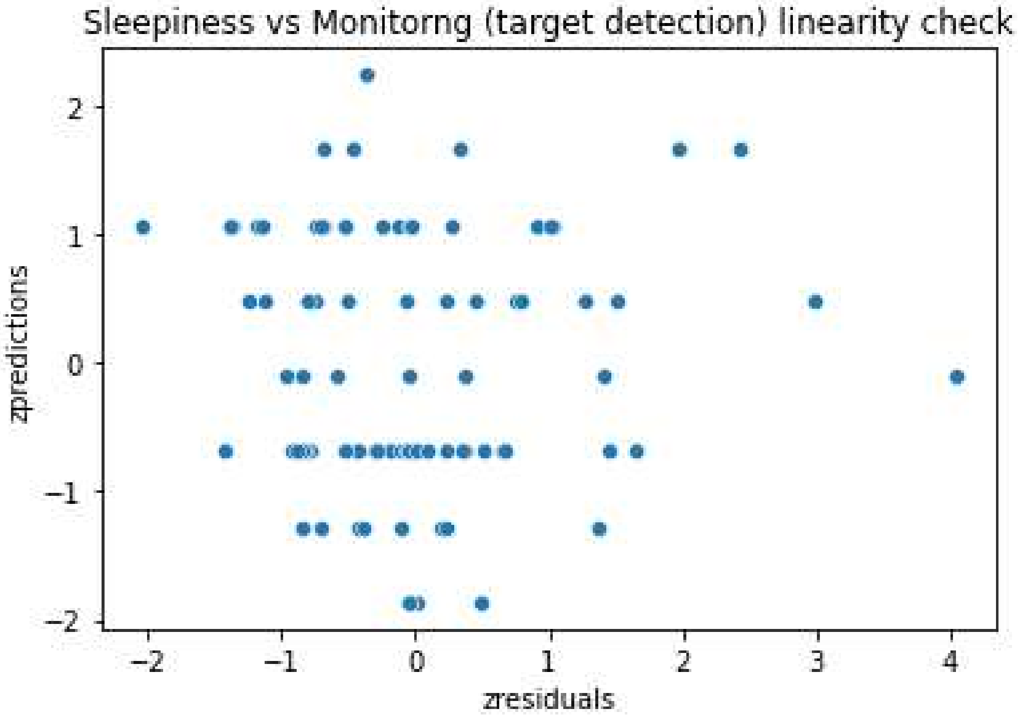

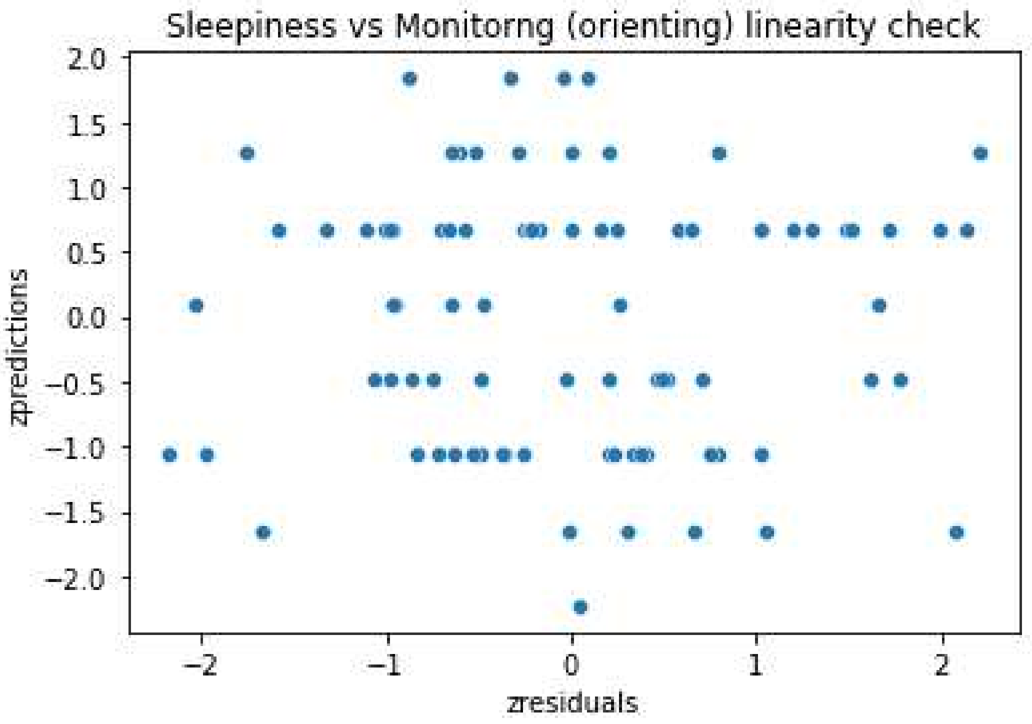
Sleepiness vs Monitorng (orienting) linearity check
zpredictions
zresiduals

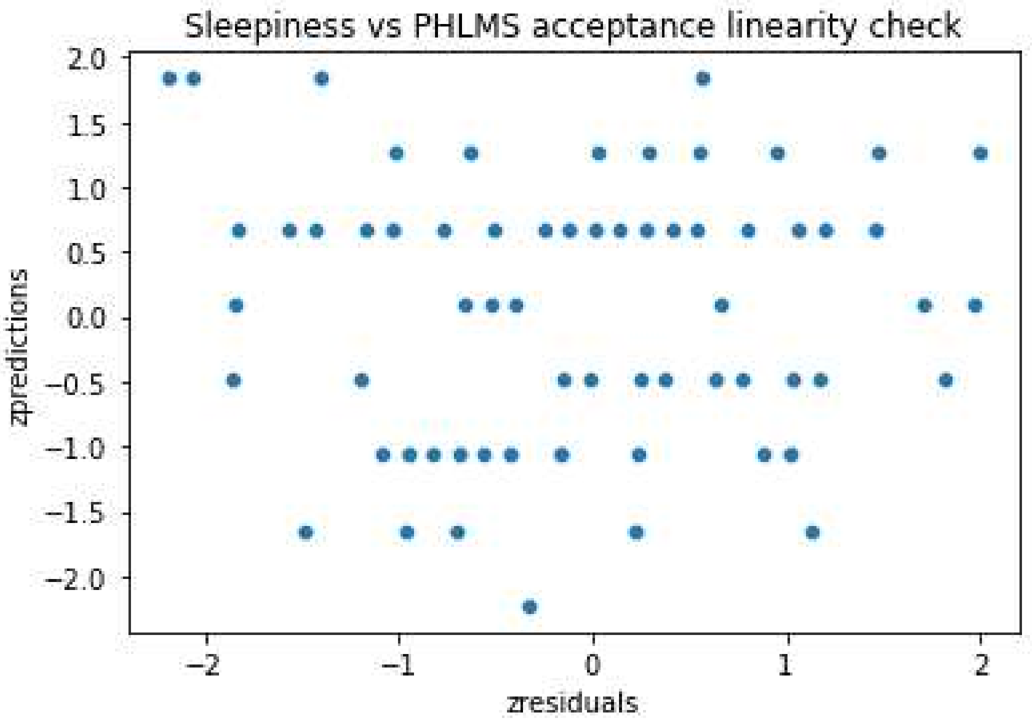
Sleepiness vs PHLMS acceptance linearity check
zpredictions
zresiduals

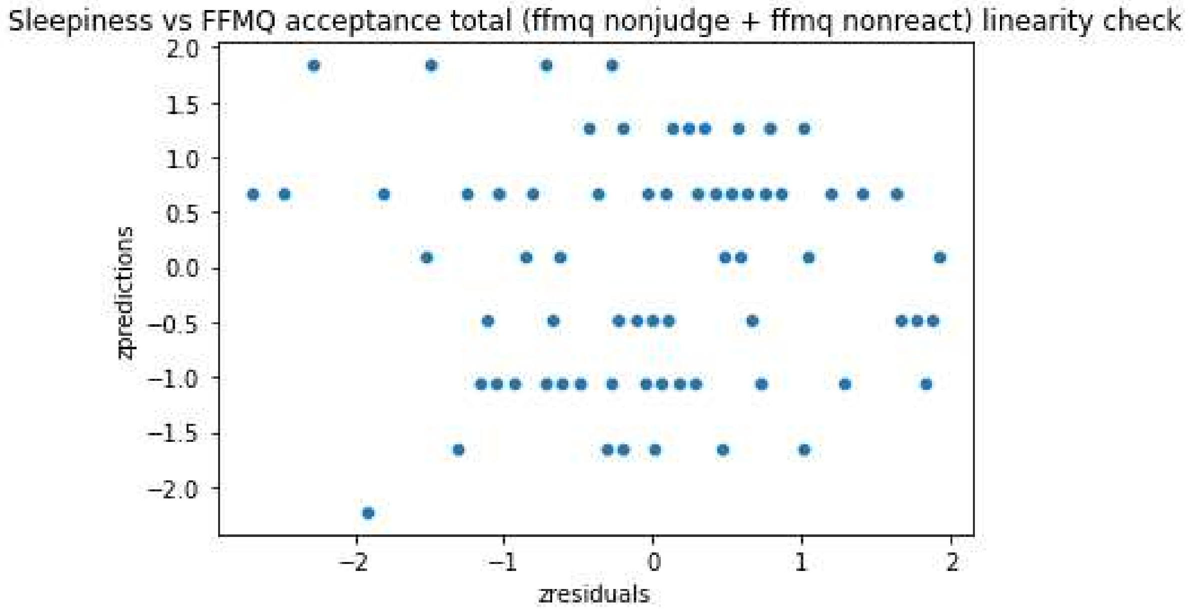
Sleepiness vs FFMQ acceptance total (ffmq nonjudge + ffmq nonreact) linearity check
zpredictions
zresiduals

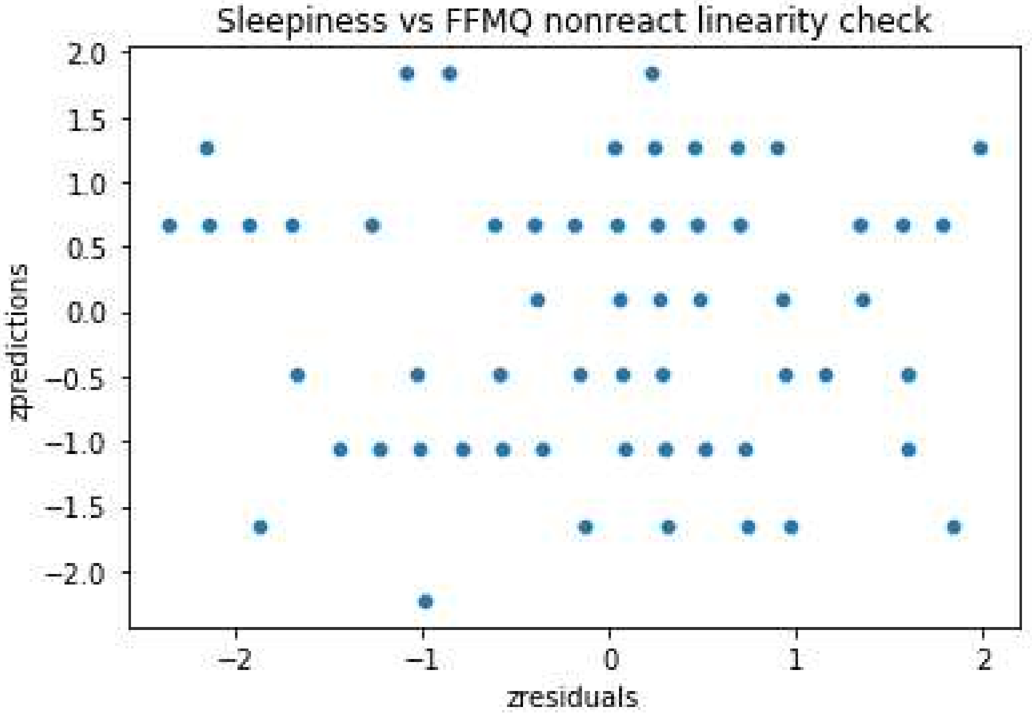
Sleepiness vs FFMQ nonreact linearity check
zpredictions
zresiduals

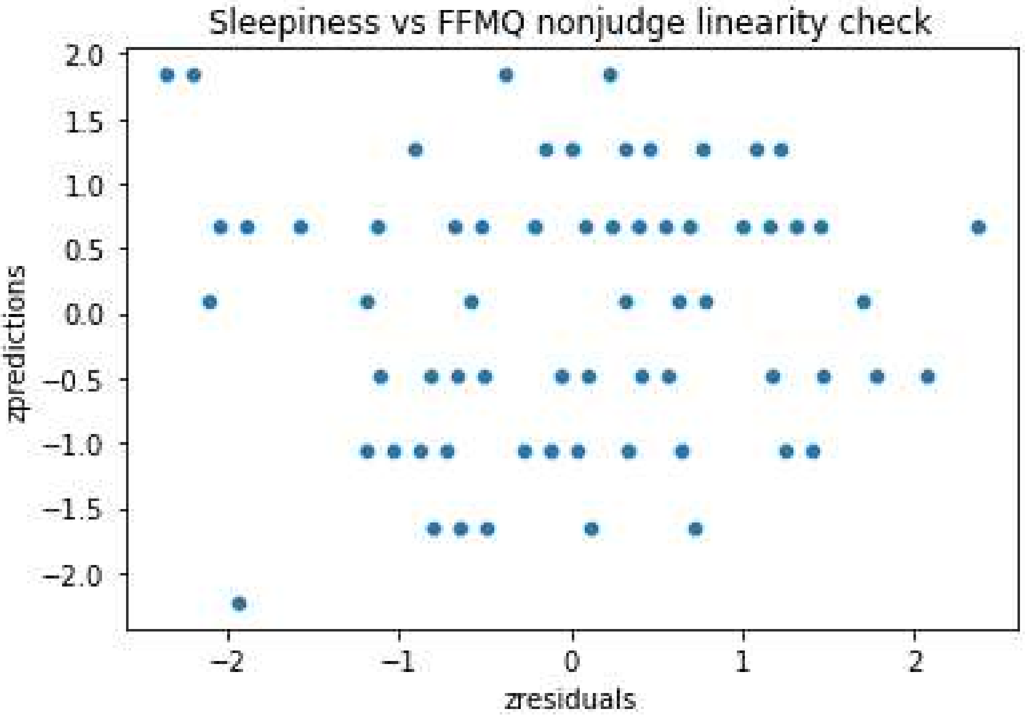
Sleepiness vs FFMQ nonjudge linearity check
zpredictions
zresiduals

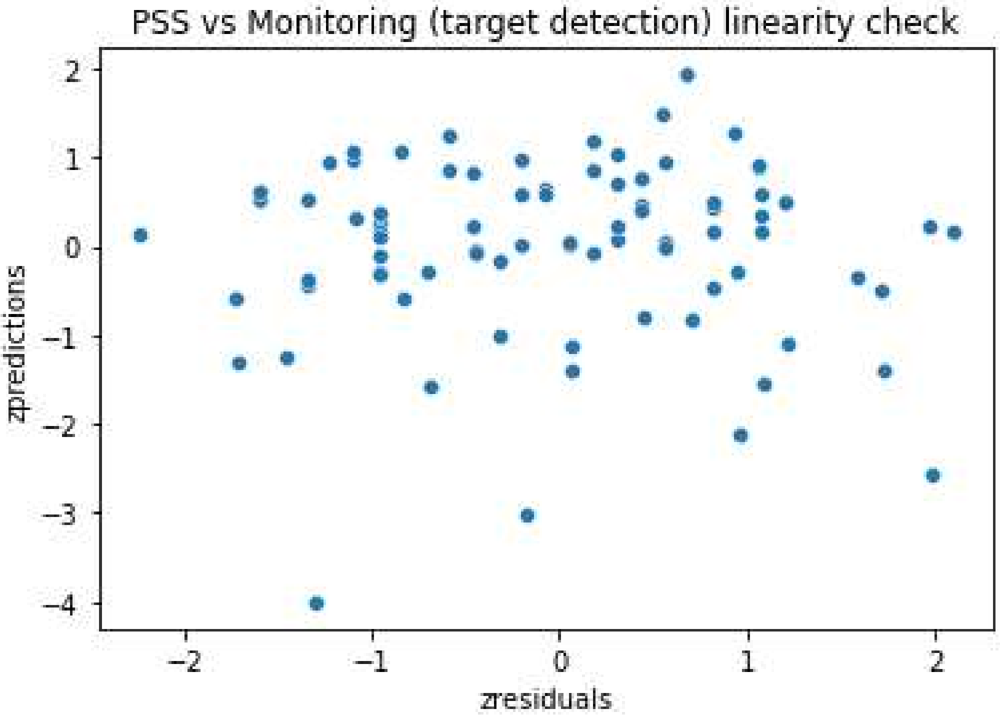
PSS vs Monitoring (target detection) linearity check
zpredictions
zresiduals

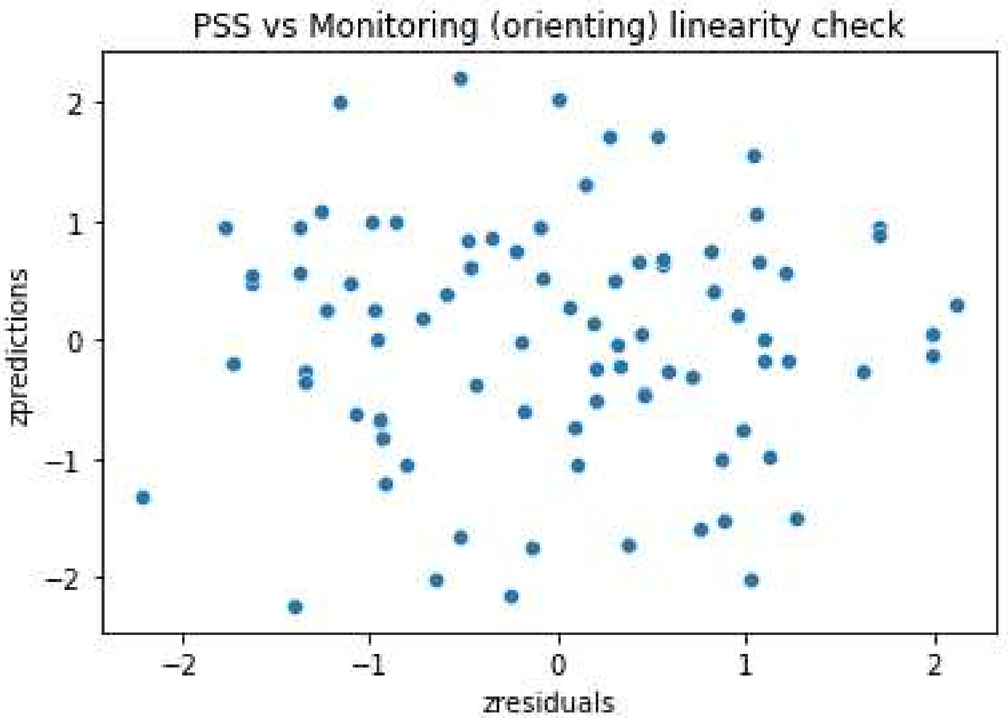
PSS vs Monitoring (orienting) linearity check
zpredictions
zresiduals
2
1
0
−1
−2
−2
−1
0
1
2

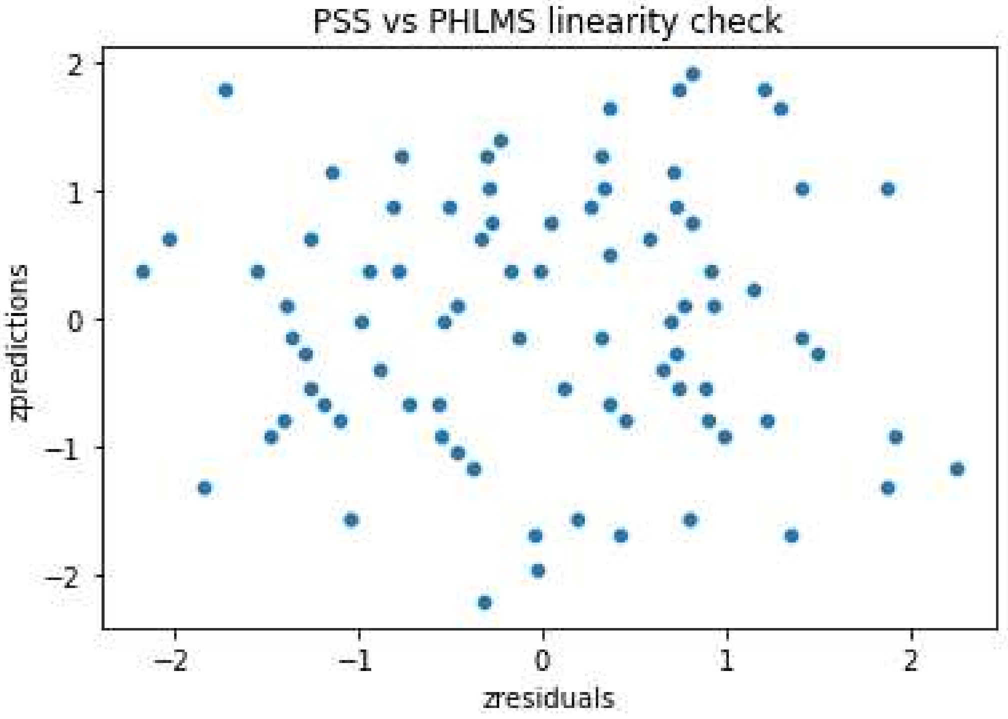
PSS vs PHLMS linearity check
zpredictions
zresiduals
2
1
0
−1
−2
−2
−1
0
1
2

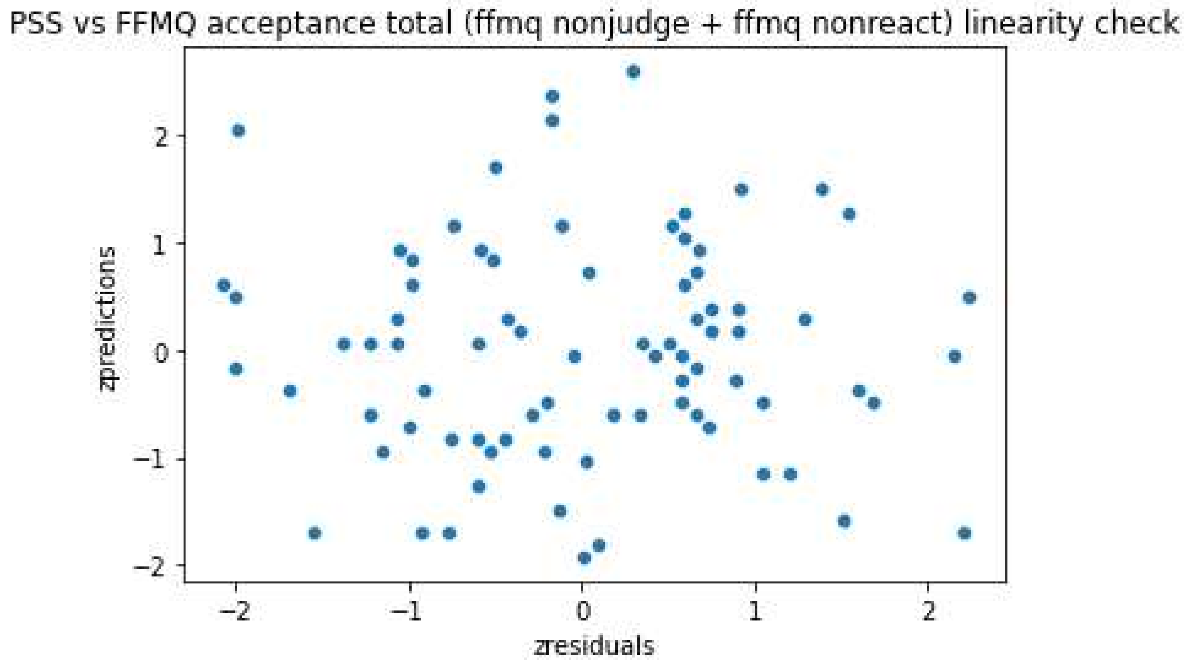

PSS vs FFMQ acceptance total (ffmq nonjudge + ffmq nonreact) linearity check
zpredictions
zresiduals

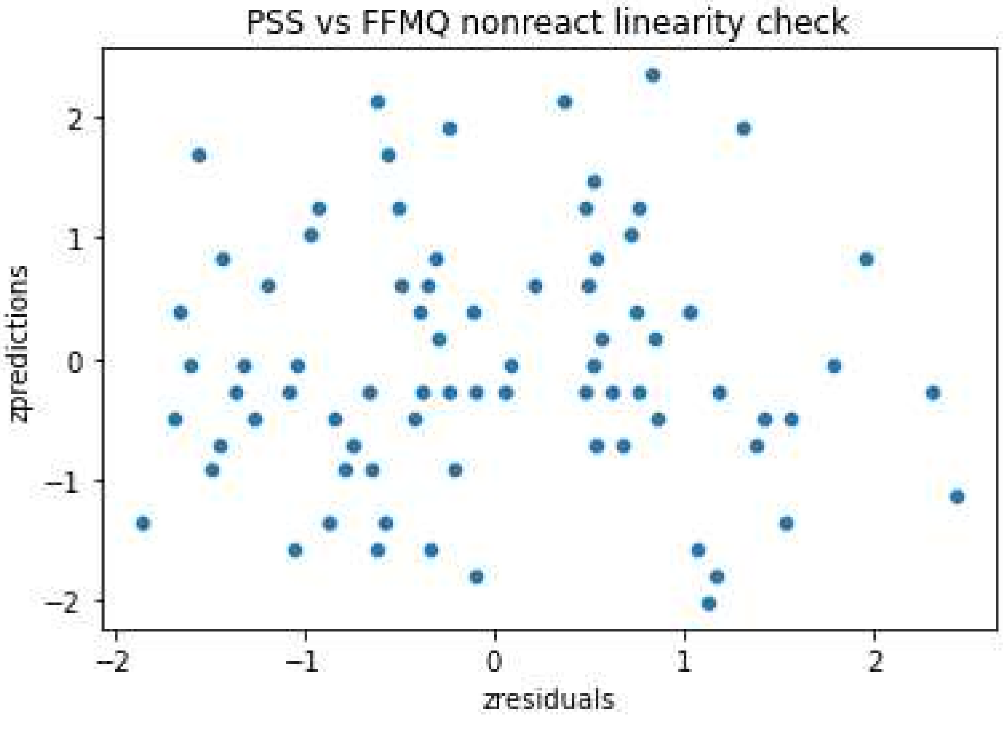

PSS vs FFMQ nonreact linearity check
zpredictions
zresiduals

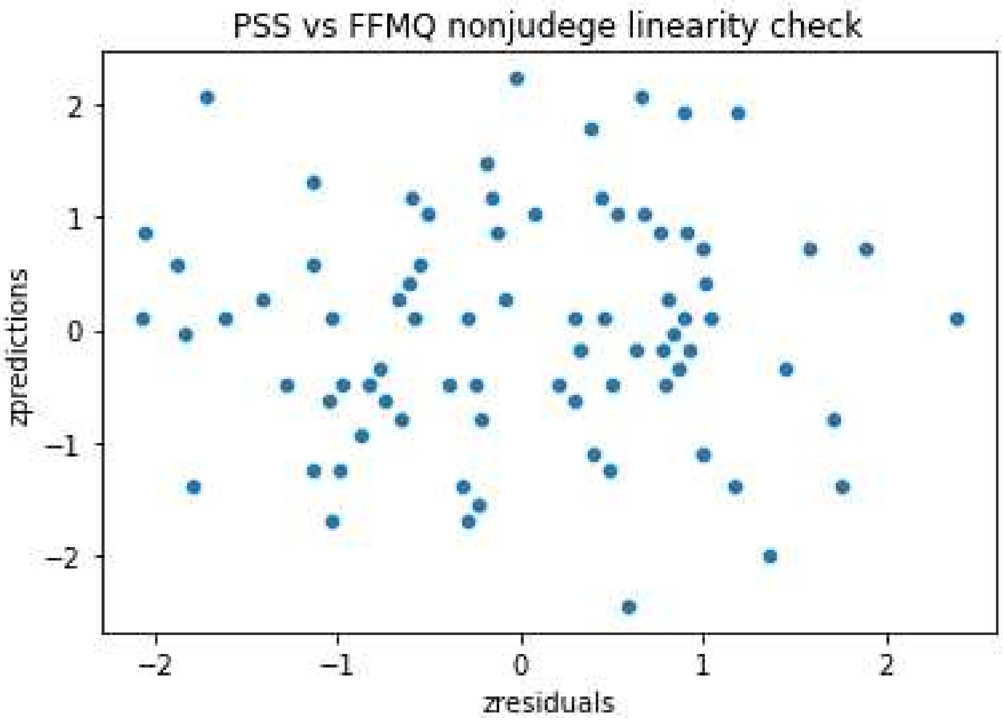

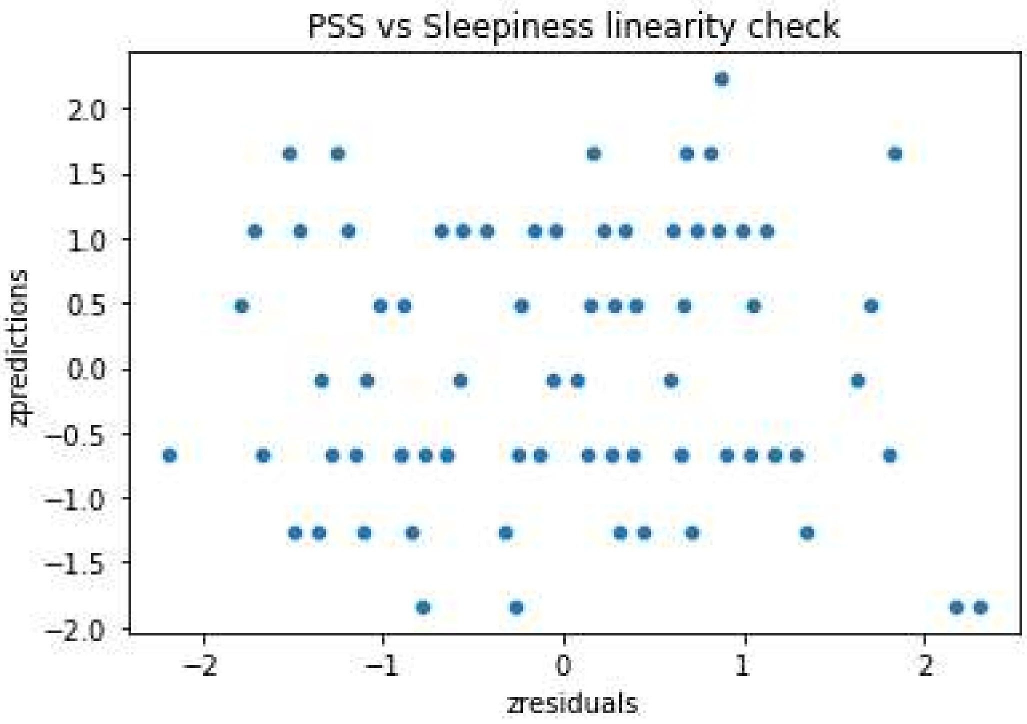

Moderation analyses full results and assumption checks. S3.

Model 1: Orienting X Acceptance

Assumption Checks

Durbin–Watson Test for Autocorrelation

Autocorrelation	DW Statistic	p
-0.0577	2.11	0.578

[3]

Collinearity Statistics

	VIF	Tolerance
ant_orienting_centered_RT	1.00	0.999
ffmq_acceptancetotal_centered	1.02	0.979
orientXaccept	1.02	0.980

[3]

Q-Q Plot

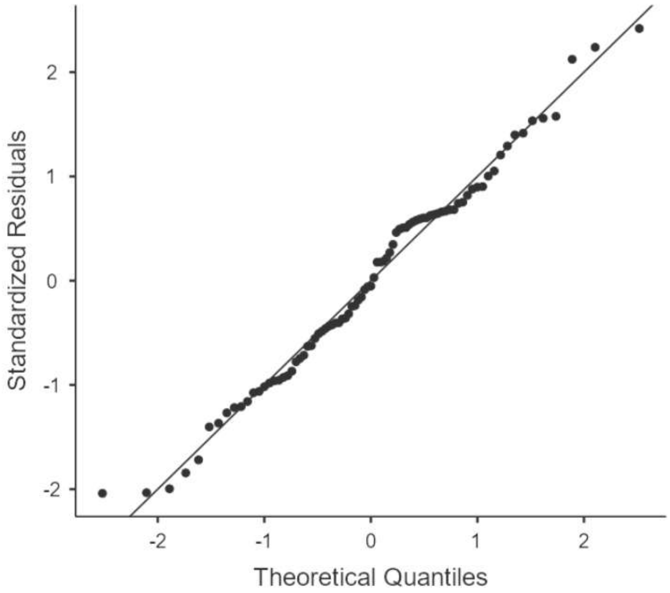

Residuals Plots

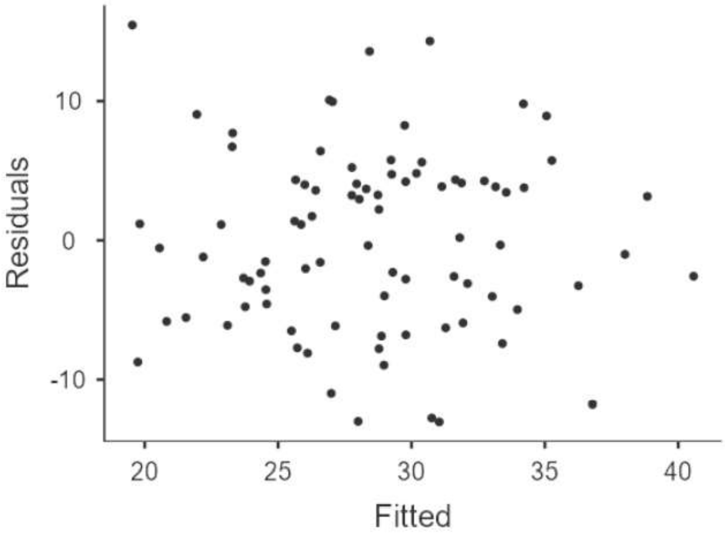

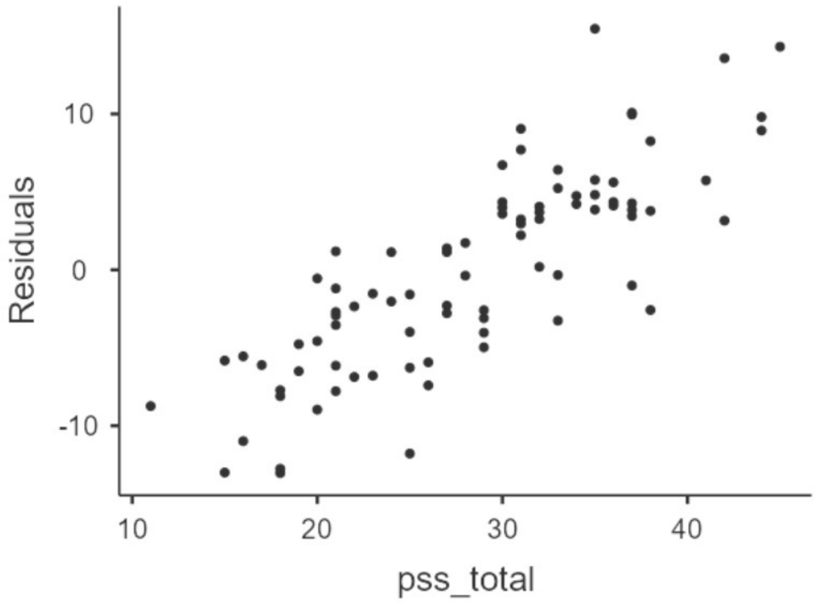
Residuals
10
0
-10
10
20
30
40
pss_total

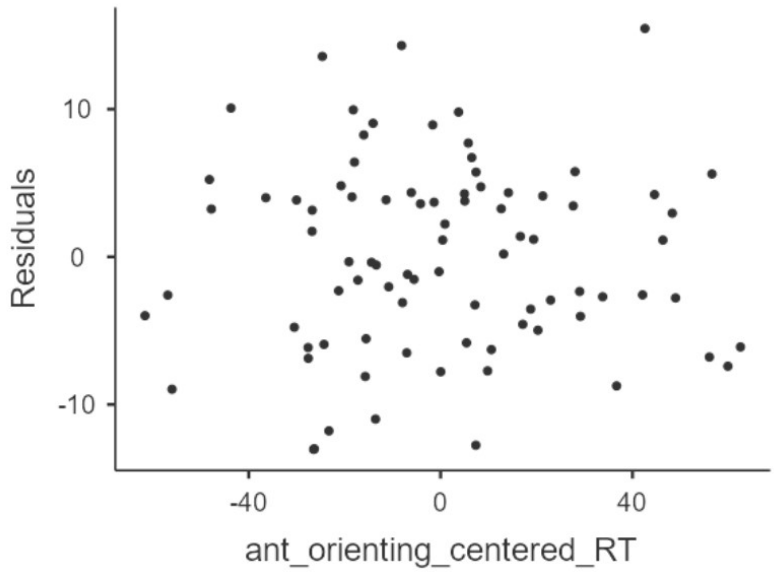
Residuals
10
0
-10
-40
0
40
ant_orienting_centered_RT

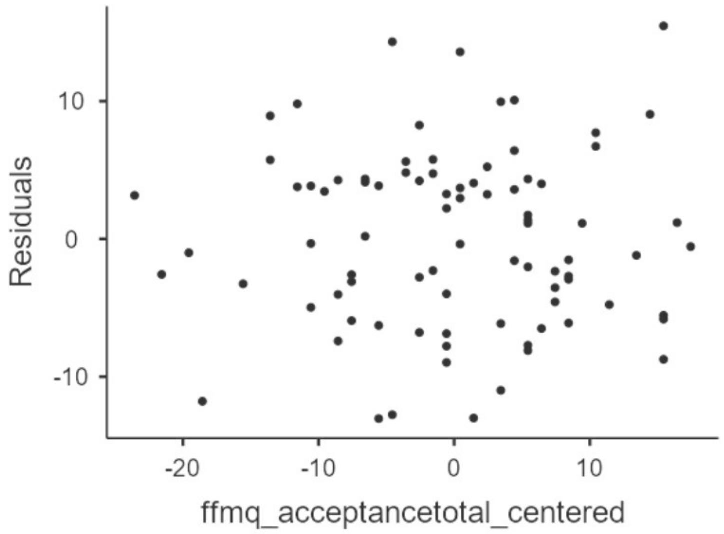

Residuals
10
0
-10
-20
-10
0
10
ffmq_acceptancetotal_centered

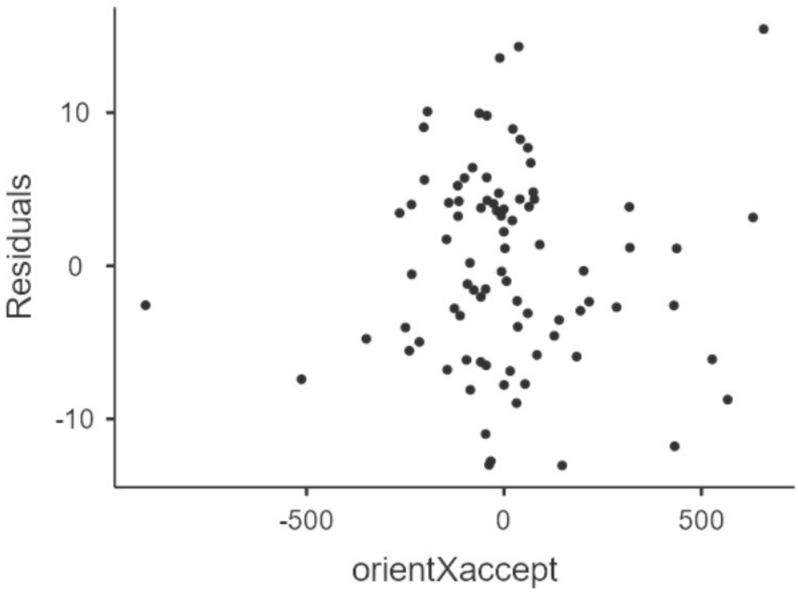

Residuals
10
0
-10
-500
0
500
orientXaccept

Model 2: Target detection X Acceptance

Assumption Checks

Durbin–Watson Test for Autocorrelation

Autocorrelation	DW Statistic	p
-0.0357	2.06	0.762

[3]

Collinearity Statistics

	VIF	Tolerance
ant_targetdetection_centered_RT	1.25	0.799
ffmq_acceptancetotal_centered	1.06	0.940
tdetectXaccept	1.18	0.846

[3]

Q-Q Plots

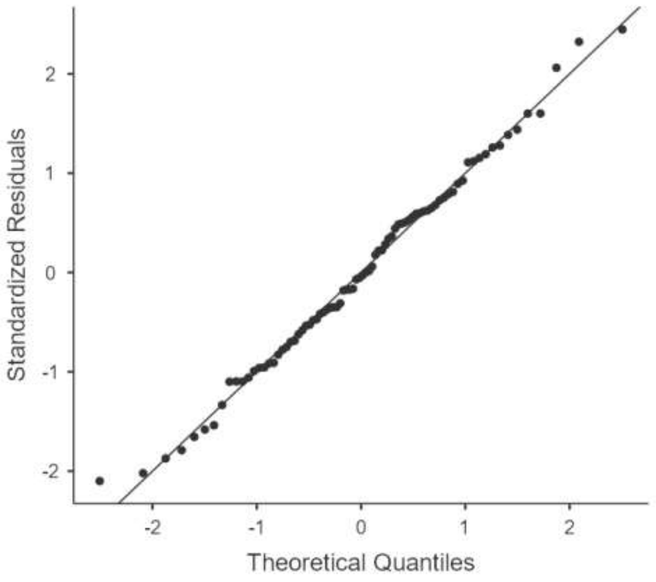

Residuals Plots

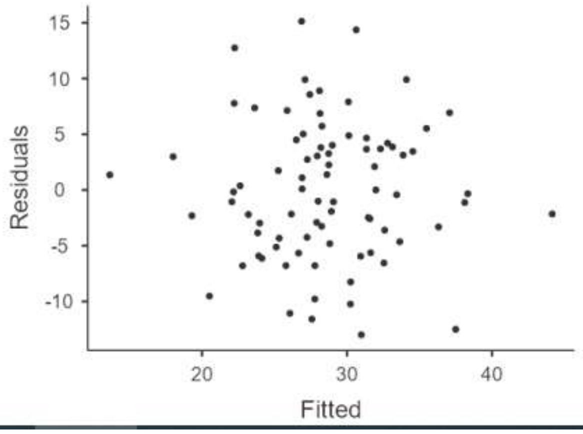

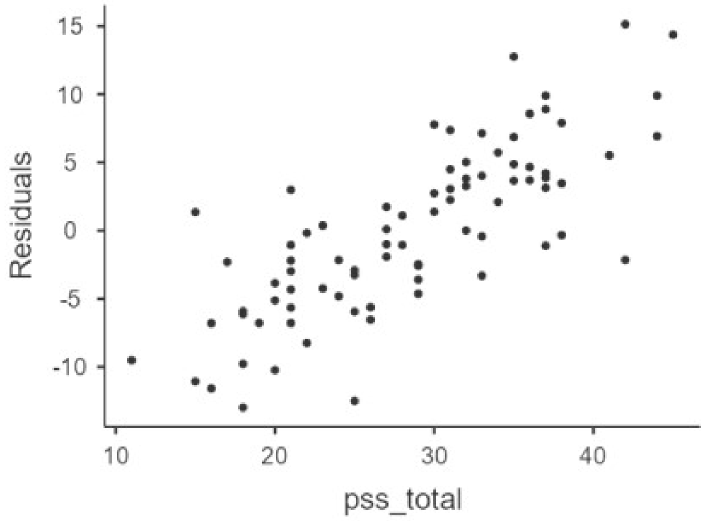
Residuals
pss_total

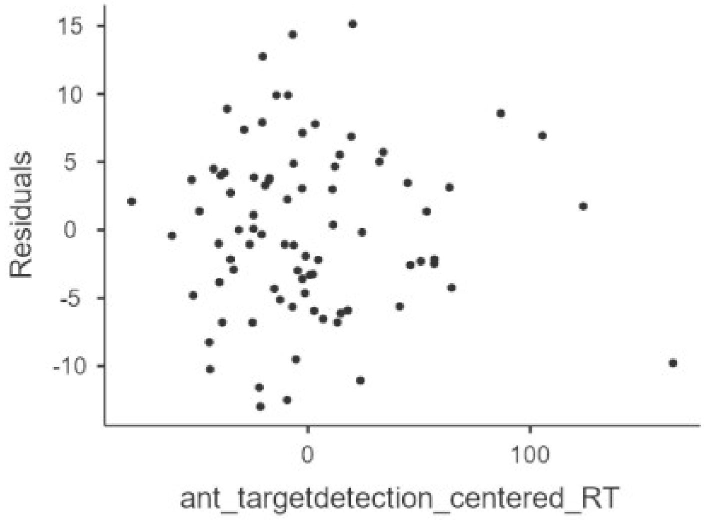
Residuals
ant_targetdetection_centered_RT

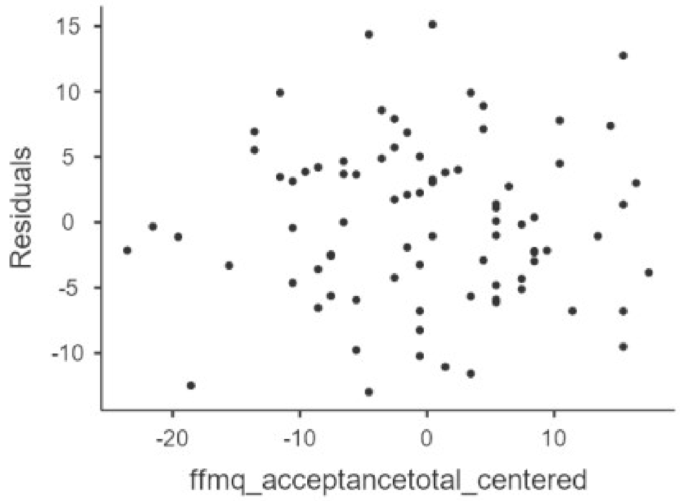

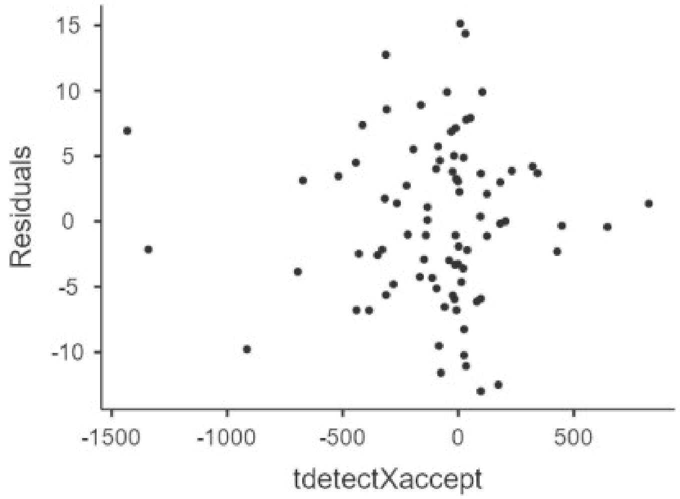

Moderation models exploring the effect of acceptance on the relationship between monitoring (i.e., orienting and target detection – separate models) on chronic stress. S4.

Below we present the results of six moderation models. The first two employ the non-judgement subscale of the FFMQ as a measure of acceptance, the second two employ the non-react subscale of the FFMQ as a measure of acceptance, and the last two employ the PHLMS acceptance subscale as a measure of acceptance. The results of these supplementary analyses are similar to the ones obtained using the combined acceptance subscales of the FFMQ as acceptance measure. That is, we found no interaction effect of orienting and acceptance, but there is an interaction between target detection and acceptance. This interaction follows the same trend of the one reported in the main text. The only exception are the analyses conducted using the FFMQ non-react subscale as a measure of acceptance. In this case, we found no interaction between the FFMQ non-react subscale and target detection.

1) **Moderation models employing the FFMQ non-judgement subscale as measure of acceptance.**

Model 1 - Interaction between orienting (i.e., orienting coefficient) and acceptance (i.e., non-judge FFMQ subscale):

At step one of our hierarchical multiple regression analysis, the two predictors (i.e., orienting and acceptance), explained 25% of the variance ($R^2 = 0.25$ Adj. $R^2 = 0.23$), which represented a statistically significant effect (*F* (2,82) = 13.56; *p* = < 0.001). However, the inclusion of the interaction term in step two did not contribute a statistically significant addition to the model (R^2 *change* = .009; *F change* (1, 81) = 1.03; *p* = 0.313). This does not statistically support the presence of moderation. Table S1 below reports all results for this model.

Table S1. Moderation model results for the prediction of Chronic Stress (i.e., PSS) from Orienting (i.e., orienting coefficient) moderated by Acceptance (i.e., *non-judge FFMQ subscale*):

			95% Confidence intervals						
**Predictor**	**Estimate (B)**	**SE**	**Lower**	**Upper**	**t**	**p**	**Standard Estimate (β)**	**r^2 a(b,c)**	**R^2 change**
Block 1									0.25
Orienting	-0.00347	0.02708	-0.0574	0.05041	-0.128	0.898	-0.0125	0.000144	
FFMQ non-judge	-0.5722	0.11335	-0.7977	-0.34668	-5.048	< .001***	-0.4876	0.233289	
Block 2									0.009
Orienting X FFMQ non-judge	-0.00484	0.00477	-0.0143	0.00465	-1.115	0.313	-0.0989	0.009409	

****p < .001*

Model 2: Interaction between target detection (i.e., target detection coefficient) and acceptance (i.e., non-judge FFMQ subscale):

At step one of our hierarchical multiple regression analysis, the two predictors (i.e., target detection and acceptance), explained 26% of the variance ($R^2 = 0.26$ Adj. $R^2 = 0.24$), which represented a statistically significant effect (*F* (2,79) = 13.8; *p* = < 0.001). The inclusion of the interaction term in step two contributed a statistically significant addition to the model (R^2 *change* = .037; *F change* (1, 78) = 4.13; *p* = < 0.045). This statistically supports the presence of moderation. The total variance explained by the model increased to 30% ($R^2 = 0.30$ Adj. $R^2 = 0.27$; *F* (3,78) = 11; *p* = < 0.001). Table S2 below reports all results for this model.

Table S2. Moderation model results for the prediction of Chronic Stress (i.e., PSS) from Target detection (i.e., orienting coefficient) moderated by Acceptance (i.e., non-judge FFMQ subscale):

			95% Confidence intervals						
**Predictor**	**Estimate (B)**	**SE**	**Lower**	**Upper**	**t**	**p**	**Standard Estimate (β)**	**r^2 a(b,c)**	**R^2 change**
Block 1									0.259
Target detection	-0.04239	0.02036	-0.0829	-0.00185	-2.08	0.041	-0.221	0.039204	
FFMQ non-judge	-0.65077	0.11616	-0.8820	-0.41952	-5.60	< .001***	-0.554	0.02347	
Block 2									0.037

Target detection X FFMQ non-judge	-0.00699	0.00344	-0.0138	-1.45e−4	-2.03	0.045	-0.210	0.037249

****p < .001*

As the moderation effect was found to be significant, we followed up this result with simple slopes analyses. Table S3 reports the results for simple slopes analysis and Figure S1 provides a visual representation of these analyses.

Table S3. Simple slopes analyses showing changes in the association between Chronic Stress (i.e., PSS) and Target detection (i.e., target detection coefficient) as a function of Acceptance (i.e., non-judge FFMQ subscale).

Moderator levels			95% Confidence Interval				
**ffmq_nonjudge_centered**	**Estimate**	**SE**	**Lower**	**Upper**	**df**	**t**	**p**
Mean-1·SD	0.00480	0.0242	-0.0433	0.05289	78.0	0.199	0.843
Mean	-0.04225	0.0203	-0.0827	-0.00176	78.0	-2.078	0.041
Mean+1·SD	-0.08931	0.0363	-0.1615	-0.01711	78.0	-2.463	0.016

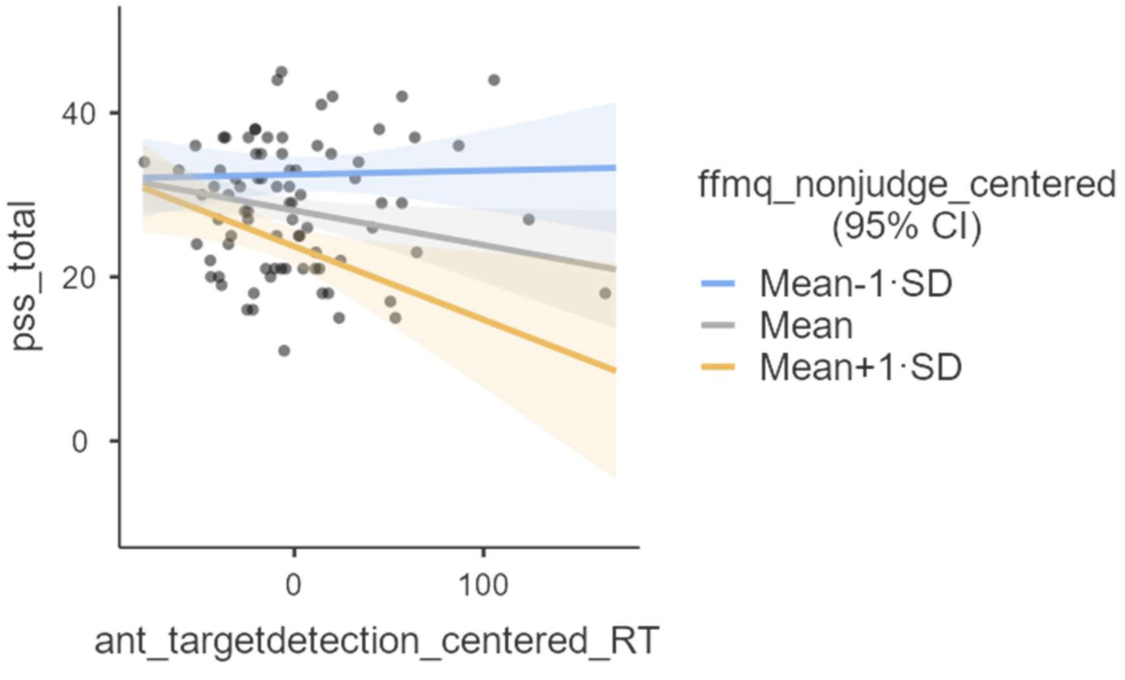

Figure S1. Visual representation of changes in the association between Chronic Stress (i.e., PSS total) and Target Detection (i.e., attention network test target detection reaction times; Fan et al., 2002), as a function of Acceptance (i.e., non-judge FFMQ subscale; Baer et al., 2006).

2) Moderation models employing the FFMQ non-react subscale as measure of acceptance.

Model 1 - Interaction between orienting (i.e., orienting coefficient) and acceptance (i.e., non-react FFMQ subscale):

At step one of our hierarchical multiple regression analysis, the two predictors (i.e., orienting and acceptance), explained 17.5% of the variance (R^2 = 0.175 Adj. R^2 = 0.155), which represented a statistically significant effect (F (2,82) = 8.71; p = < 0.001). However, the inclusion of the interaction term in step two did not contribute a statistically significant addition to the model (R^2 *change* = 0.000512; *F change* (1, 81) = 0.0503; p = 0.823). This does not statistically support the presence of moderation. Table S4 below reports all results for this model.

Table S4. Moderation model results for the prediction of Chronic Stress (i.e., PSS) from Orienting (i.e., orienting coefficient) moderated by Acceptance (i.e., non-react FFMQ subscale).

			95% Confidence intervals						
**Predictor**	**Estimate (B)**	**SE**	**Lower**	**Upper**	**t**	**p**	**Standard Estimate (β)**	**r^2 a(b,c)**	**R^2 change**
Block 1									0.175
Orienting	-0.01271	0.02867	-0.0698	0.0443	-0.443	0.659	-0.0456	0.002025	
FFMQ non-react	-0.70546	0.17230	-1.0483	-0.3626	-4.094	< .001***	-0.4155	0.170569	
Block 2									0.000512
Orienting X FFMQ non-react	-0.00156	0.00697	-0.0154	0.0123	-0.224	0.823	-0.0231	0.000529	

****p < .001*

Model 2: Interaction between target detection (i.e., target detection coefficient) and acceptance (i.e., non-react FFMQ subscale):

At step one of our hierarchical multiple regression analysis, the two predictors (i.e., target detection and acceptance), explained 20% of the variance (R^2 = 0.196 Adj. R^2 = 0.175), which represented a statistically significant effect (F (2,79) = 9.6; p = < 0.001). The inclusion of the interaction term in step two contributed a statistically significant addition to the model (R^2 *change* = .0115; *F change* (1, 78) = 1.13; p = 0.290). This does not statistically support the presence of moderation. Table S5 below reports all results for this model.

Table S5. Moderation model results for the prediction of Chronic Stress (i.e., PSS) from Target detection (i.e., orienting coefficient) moderated by Acceptance (i.e., non-react FFMQ subscale):

Predictor	Estimate (B)	SE	95% Confidence intervals		t	p	Standard Estimate (β)	r^2 a(b,c)	R^2 change
			Lower	Upper					
Block 1									0.196
Target detection	-0.01833	0.02009	-0.0583	0.02165	-0.913	0.364	-0.0958	0.008464	
FFMQ non-react	-0.75074	0.17570	-1.1005	-0.40096	-4.273	< .001***	-0.4364	0.185761	
Block 2									0.0115
Target detection X FFMQ non-react	-0.00510	0.00479	-0.0146	0.00444	-1.064	0.290	-0.1115	0.011449	

****p < .001*

3) Moderation models employing the PHLMS acceptance subscale as measure of acceptance.

Model 1 - Interaction between orienting (i.e., orienting coefficient) and acceptance (i.e., PHLMS acceptance subscale):

At step one of our hierarchical multiple regression analysis, the two predictors (i.e., orienting and acceptance), explained 31% of the variance (R^2 = 0.306 Adj. R^2 = 0.289), which represented a statistically significant effect (*F* (2,82) = 18; *p* = < 0.001). However, the inclusion of the interaction term in step two did not contribute a statistically significant addition to the model (*R^2 change* = .002; *F change* (1, 81) = 0.283; *p* = 0.6). This does not statistically support the presence of moderation. Table S6 below reports all results for this model.

Table S6. Moderation model results for the prediction of Chronic Stress (i.e., PSS) from Orienting (i.e., orienting coefficient) moderated by Acceptance (i.e., PHLMS acceptance subscale).

Predictor	Estimate (B)	SE	95% Confidence intervals		t	p	Standard Estimate (β)	r^2 a(b,c)	R^2 change
			Lower	Upper					
Block 1									0.306
Orienting	0.01101	0.02599	-0.04069	0.06272	0.424	0.673	0.0395	0.001521	
PHLMS acceptance	-0.54746	0.09533	-0.73714	-0.35779	-5.743	< .001***	-0.5459	0.281961	
Block 2									0.00242
Orienting X PHLMS acceptance	-0.00180	0.00339	-0.00855	0.00494	-0.532	0.596	-0.0504	0.002401	

****p < .001*

Model 2: Interaction between target detection (i.e., target detection coefficient) and acceptance (i.e., PHLMS acceptance subscale):

At step one of our hierarchical multiple regression analysis, the two predictors (i.e., target detection and acceptance), explained 32% of the variance (R^2 = 0.319 Adj. R^2 = 0.301), which represented a

statistically significant effect (*F* (2,79) = 18.5; *p* = < 0.001). The inclusion of the interaction term in step two contributed a statistically significant addition to the model (R^2 *change* = .05; *F change* (1, 78) = 6.59; *p* = < 0.012). This statistically supports the presence of moderation. The total variance explained by the model increased to 37% (R^2 = 0.372 Adj. R^2 = 0.348; *F* (3,78) = 15.4; *p* = < 0.001). Table S7 below reports all results for this model.

Table S7. Moderation model results for the prediction of Chronic Stress (i.e., PSS) from Target detection (i.e., orienting coefficient) moderated by Acceptance (i.e., PHLMS acceptance subscale):

			95% Confidence intervals						
**Predictor**	**Estimate (B)**	**SE**	**Lower**	**Upper**	**t**	**p**	**Standard Estimate (β)**	**r^2 a(b,c)**	**R^2 change**
Block 1									0.319
Target detection	-0.03861	0.01864	-0.0757	-0.00151	-2.07	0.042	-0.202	0.034596	
PHLMS acceptance	-0.58102	0.09132	-0.7628	-0.39921	-6.36	< .001***	-0.581	0.326041	
Block 2									0.0531
Target detection X PHLMS acceptance	-0.00623	0.00243	-0.0111	-0.00140	-2.57	0.012	-0.246	0.0529	

****p < .001*

As the moderation effect was found to be significant, we followed up this result with simple slopes analyses. Table S8 reports the results for simple slopes analysis and Figure S2 provides a visual representation of these analyses.

Table S8. Simple slopes analyses showing changes in the association between Chronic Stress (i.e., PSS) and Target detection (i.e., target detection coefficient) as a function of Acceptance (i.e., PHLMS acceptance subscale).

Moderator levels			95% Confidence Interval				
**phlms_acceptance**	**Estimate**	**SE**	**Lower**	**Upper**	**df**	**t**	**p**
Mean-1·SD	0.0103	0.0215	-0.0326	0.05313	78.0	0.477	0.635
Mean	-0.0390	0.0187	-0.0762	-0.00177	78.0	-2.085	0.040
Mean+1·SD	-0.0882	0.0311	-0.1502	-0.02620	78.0	-2.832	0.006

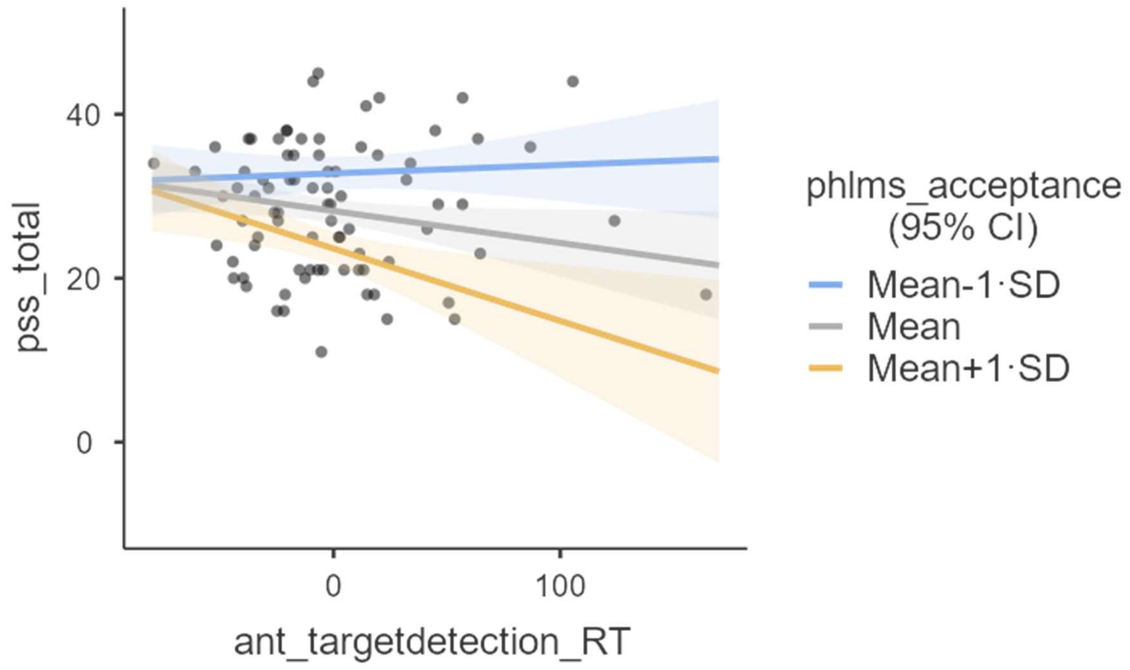

Figure S2. Visual representation of changes in the association between Chronic Stress (i.e., PSS total) and Target Detection (i.e., attention network test target detection reaction times; Fan et al., 2002), as a function of Acceptance (i.e., PHLMS acceptance subscale; Cardaciotto et al., 2008).
